# Effective Menin inhibitor-based combinations against AML with MLL rearrangement or NPM1 mutation (NPM1c)

**DOI:** 10.1038/s41408-021-00603-3

**Published:** 2022-01-11

**Authors:** Warren Fiskus, Steffen Boettcher, Naval Daver, Christopher P. Mill, Koji Sasaki, Christine E. Birdwell, John A. Davis, Koichi Takahashi, Tapan M. Kadia, Courtney D. DiNardo, Qi Jin, Yuan Qi, Xiaoping Su, Gerard M. McGeehan, Joseph D. Khoury, Benjamin L. Ebert, Kapil N. Bhalla

**Affiliations:** 1 The University of Texas M.D. Anderson Cancer Center, Houston, TX 77030 USA; 2 University of Zurich and University Hospital Zurich, CH-8091 Zurich, Switzerland; 3 Syndax Pharmaceuticals, Inc., Waltham, MA 02451 USA; 4 Howard Hughes Medical Institute, Dana-Farber Cancer Institute, Boston, MA 02115 USA

**Keywords:** Acute myeloid leukaemia, Targeted therapies

## Abstract

Treatment with Menin inhibitor (MI) disrupts the interaction between Menin and MLL1 or MLL1-fusion protein (FP), inhibits HOXA9/MEIS1, induces differentiation and loss of survival of AML harboring MLL1 re-arrangement (r) and FP, or expressing mutant (mt)-NPM1. Following MI treatment, although clinical responses are common, the majority of patients with AML with MLL1-r or mt-NPM1 succumb to their disease. Pre-clinical studies presented here demonstrate that genetic knockout or degradation of Menin or treatment with the MI SNDX-50469 reduces MLL1/MLL1-FP targets, associated with MI-induced differentiation and loss of viability. MI treatment also attenuates BCL2 and CDK6 levels. Co-treatment with SNDX-50469 and BCL2 inhibitor (venetoclax), or CDK6 inhibitor (abemaciclib) induces synergistic lethality in cell lines and patient-derived AML cells harboring MLL1-r or mtNPM1. Combined therapy with SNDX-5613 and venetoclax exerts superior in vivo efficacy in a cell line or PD AML cell xenografts harboring MLL1-r or mt-NPM1. Synergy with the MI-based combinations is preserved against MLL1-r AML cells expressing FLT3 mutation, also CRISPR-edited to introduce mtTP53. These findings highlight the promise of clinically testing these MI-based combinations against AML harboring MLL1-r or mtNPM1.

## Introduction

MLL1 (KMT2A) is a large (3696 amino acids) transcriptional regulator and a histone-lysine-N-methyltransferase [1, 2]. N-terminal 1400 amino acids of MLL1 act as a transcription factor containing Menin binding domains (MBD), and the C-terminal SET domain acts as a histone methyltransferase that mediates histone H3 lysine 4 trimethylation [1, 2]. Encoded by the MEN1 gene, Menin is a 610 amino acid, scaffold protein [3, 4]. Menin binds to MBD within the N-terminal 1–40 residues of MLL1 and forms a Menin-MLL1-LEDGF ternary complex, which tethers MLL1 to chromatin [2, 5]. Menin–KMT2A complex has a critical role in regulating HOX genes cluster involved in embryonic development and hematopoiesis, including the leukemogenic homeobox A9 (HOXA9) and its co-factor MEIS1 in myeloid stem progenitor cells [6–8]. HOXA9 functions as a pioneer factor and along with its co-factor MEIS1 recruits CEBPα and MLL3/MLL4 to reprogram the enhancer landscape to promote leukemogenesis [9]. MLL1 knockout (KO) is embryonic lethal, but conditional KO undermines the self-renewal of hematopoietic stem cells (HSCs) [10, 11]. In MLL1-rearranged (MLL1-r) AML, N-terminus of the MLL1 gene is fused to the C-terminus of any of over 80 fusion partners, including AF4, AF9, ENL, and ELL, which are part of and recruit the super elongation complex (SEC) (including AFF1/4 and pTEFb) and DOT1L to induce H3K4Me3 and H3K79Me2 marks on active chromatin [2, 12–15]. MLL1 fusion protein (MLL-FP) binds to gene targets largely different from wild-type MLL1 and dysregulates HOXA9, MEIS1, PBX3, MEF2C, and CDK6 [2, 16, 17]. Conditional KO of MEN1 prevents MLL1-r AML [2, 5, 6]. In AML with mutant (mt) NPM1 (NPM1c), MLL1 is the main oncogenic regulator of HOXA9, MEIS1, and FLT3, promoting self-renewal of myeloid progenitor cells [2, 18–20]. Treatment with orally bioavailable, investigational or clinical drug candidate Menin inhibitor (MI), SNDX-50469, or SNDX-5613 disrupts binding of Menin to its binding pocket in MLL1/2 and MLL1-FP [2, 17]. This evicts Menin from the chromatin and reduces MLL1/2 and MLL1-FP binding to their targets, thereby repressing HOXA9, MEIS1, PBX3, MEF2C, FLT3, and CDK6 [2, 17, 20]. Importantly, treatment with MI leads to differentiation and apoptosis of AML cells expressing MLL-FP or NPM1c [2, 17, 20–22]. Present studies further elucidate the effects of MI treatment on chromatin accessibility, active enhancers/promoters, as well as on transcriptome/proteome that correlate with MI-induced anti-AML efficacy in cell lines and patient-derived (PD) AML blast progenitor cells (BPCs) of these AML models. In phase I/II trials, monotherapy with MIs is well tolerated and has achieved objective remissions in patients with previously treated relapsed/refractory AML harboring MLL1-r or NPM1c [2, 23]. However, the majority of patients either fail to respond or eventually relapse [23]. A minority of MLL1-r AML also exhibit a co-mutation in TP53, which is known to confer therapy resistance and poor outcome in AML [24, 25]. This underscores the need to pre-clinically develop synergistic combinations that would exert superior in vitro and in vivo efficacy against PD AML cells harboring MLL-FP or NPM1c, including those that also express mutant TP53. Present studies demonstrate that MI-based combination with BCL2 or CDK6 inhibitor exerted synergistic lethal activity against PD AML cells harboring MLL-FP or NPM1c. The combination of MI and BCL2 inhibitor also synergistically induced lethality against AML cells co-expressing MLL-FP and mutant TP53. Finally, compared to treatment with each agent alone, co-treatment with SNDX-5613 and the BCL2 inhibitor venetoclax significantly reduced AML burden and improved overall survival of immune-depleted mice engrafted with AML cells expressing MLL-FP or NPM1c.

## Materials and methods

### Reagents and antibodies

SNDX-50469, venetoclax, gilteritinib, cobimetinib, etoposide, and abemaciclib were obtained from MedChem Express (Monmouth Junction, NJ). DTAG-13 (Cat. No. 6605) was obtained from Tocris/Bio-Techne (Minneapolis, MN). SNDX-5613 was obtained from Syndax Pharmaceuticals Inc. (Waltham, MA) under a material transfer agreement. All compounds were prepared as 10 mM stocks in 100% DMSO and frozen at −80 °C in 5–10 µL aliquots to allow for single use, thus avoiding multiple freeze-thaw cycles that could result in compound decomposition and loss of activity. Anti-c-Myc [RRID:AB_1903938], anti-MCL1 [RRID:AB_2799149], anti-BIM [RRID:AB_1030947], anti-BAK [RRID:AB_10828597], anti-BAX [RRID:AB_10557411], anti-PUMA [RRID:AB_2797920], anti-Bcl-xL [RRID:AB_10695729], anti-Menin [RRID:AB_10858216], anti-β-Tubulin [#86298], anti-MEF2C [RRID:AB_10548759], anti-p-ERK1/2 [RRID:AB_331772], ERK1/2 [RRID:AB_390779], p-S6 [RRID:AB_916156], total S6 [RRID:AB_331355], p-Rb [RRID:AB_11178658], total Rb [RRID:AB_823629], and p16 [RRID:AB_2799960] antibodies were obtained from Cell Signaling Technologies (Beverly, MA). Anti-HA-tag [RRID:AB_444303], Anti-MEIS1 [RRID:AB_776272], anti-FLT3 [ab245116], anti-HOXA9 [ab140631], anti-PBX3 [RRID:AB_10858991], and anti-CD11b [RRID:AB_2650514] antibodies were obtained from Abcam (Cambridge, MA). Anti-BFL1 [# ABC490] antibody was obtained from Millipore/Sigma (Burlington, MA). Anti-CDK6 [RRID:AB_10610066], anti-BCL2 [RRID:AB_626733], anti-NOXA [RRID:AB_784877], anti-GAPDH [RRID:AB_627679] and anti-β-Actin [RRID:AB_626630] antibodies were obtained from Santa Cruz Biotechnologies (Santa Cruz, CA). Anti-p27 [RRID:AB_397636] antibody was obtained from BD Transduction Labs (Franklin Lakes, NJ).

### Cell lines and cell culture

MOLM13 [DSMZ Cat# ACC-554, RRID: CVCL_2119] and OCI-AML3 [DSMZ Cat# ACC-582, RRID: CVCL_1844] cells were obtained from the DSMZ. MV4-11 [ATCC Cat# CRL-9591, RRID: CVCL_0064], cells were obtained from the ATCC (Manassas, VA). MOLM13 cells with isogenic TP53 mutations [R175H, R248Q, and TP53-KO] were a gift from Dr. Benjamin L. Ebert (Dana Farber Cancer Center, Boston, MA). HEK-293T cells were obtained from the Characterized Cell Line Core Facility at M.D. Anderson Cancer Center, Houston TX. All experiments with cell lines were performed within 6 months after thawing or obtaining from ATCC or DSMZ. The cell lines were also authenticated in the Characterized Cell Line Core Facility at M.D. Anderson Cancer Center, Houston TX. MOLM13 and OCI-AML3 cells were cultured in RPMI-1640 media with 20% fetal bovine serum (FBS) and 1% penicillin/streptomycin. MV4-11 cells were cultured in ATCC-formulated IMDM media with 20% FBS and 1% penicillin/streptomycin. HEK293T cells were cultured in high-glucose-formulated DMEM media with 10% FBS, 1% penicillin/streptomycin, and 1% glutamine. Logarithmically growing, mycoplasma-negative cells were utilized for all experiments. Following drug treatments, cells were washed free of the drug(s) prior to the performance of the studies described.

### Cell line authentication

The cell lines utilized in these studies were authenticated in the Characterized Cell Line Core Facility at M.D. Anderson Cancer Center, Houston TX utilizing STR profiling.

### Assessment of percentage non-viable cells

Following designated treatments, cultured cell lines or PD-AML cells were washed with 1× phosphate-buffered saline, stained with TO-PRO-3 iodide (Life Technologies, Carlsbad, CA), and analyzed by flow cytometry on a BD Accuri CFlow-6 flow cytometer (BD Biosciences, San Jose, CA). We used matrix dosing of agents in combinations to allow synergy assessment utilizing the SynergyFinder V2 online web application tool (http://synergyfinder.fimm.fi/). Delta synergy scores were determined by the ZIP method.

### RNA isolation and quantitative polymerase chain reaction

Following the designated treatments, total RNA was isolated from cultured or PD AML cells utilizing a PureLink RNA Mini kit from Ambion, Inc. (Austin, TX) and reverse transcribed with a High-Capacity Reverse Transcription kit from Life Technologies (Carlsbad, CA). Quantitative real-time PCR analysis for the expression of target genes was performed on cDNA using TaqMan probes and a TaqMan Universal PCR Mastermix from Applied Biosystems (Foster City, CA). Relative mRNA expression was normalized to the expression of GAPDH and compared to the untreated cells.

## Results

### CRISPR–Cas9-mediated Menin knockout or dTAG-13-induced degradation of Menin increases sensitivity to venetoclax or abemaciclib in AML cells with MLL1-r

**Fig. 1:**
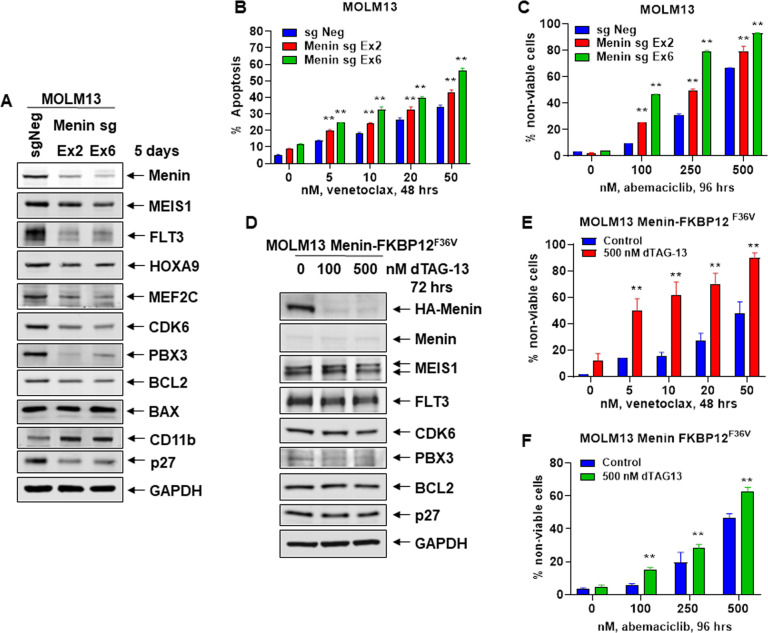
Depletion or degradation of Menin increases sensitivity to treatment with venetoclax or abemaciclib in AML cells. **A** MOLM13 was transfected with sgRNA against Exon 2 or Exon 6 of Menin or a negative control sgRNA (sgNeg) and incubated for 5 days. Then, total cell lysates were prepared and immunoblot analyses were conducted. The expression levels of GAPDH served as the loading control. **B** MOLM13 cells were transfected with sgNeg or sg Menin Ex 2 or Ex 6 and incubated for 72 h. Then, cells were treated with the indicated concentrations of venetoclax for 48 h. The % of annexin V-positive, apoptotic cells were determined by flow cytometry. Mean of two independent experiments performed in duplicate ±S.D. **p < 0.01 compared to sgNeg-transfected MOLM13 cells (determined by a two-tailed, unpaired *t* test). **C** MOLM13 cells were transfected with sgNeg or sg Menin Ex 2 or Ex 6 and incubated for 72 h. Following this, cells were treated with the indicated concentrations of abemaciclib for 96 h. The % of TO-PRO-3 iodide-positive, non-viable cells were determined by flow cytometry. Mean of two independent experiments performed in duplicate ±S.D. **p < 0.01 compared to sgNeg-transfected MOLM13 cells (determined by a two-tailed, unpaired *t* test). **D** MOLM13-Menin-FKBP12^F36V^ cells were treated with the indicated concentrations of dTAG-13 for 72 h. At the end of treatment, cell lysates were prepared and immunoblot analyses were conducted for HA-tagged Menin, endogenous Menin, MEIS1, FLT3, CDK6, PBX3, BCL2, and p27. The expression levels of GAPDH served as the loading control. **E** MOLM13-Menin-FKBP12^F36V^ cells were treated with the indicated concentrations of venetoclax for 48 h alone or co-treated with 500 nM of dTAG-13. The % of TO-PRO-3 iodide-positive, non-viable cells were determined by flow cytometry. Mean of two independent experiments performed in duplicate ±S.D. **p < 0.01 compared to MOLM13-Menin-FKBP12^F36V^ cells not treated with dTAG-13 (determined by a two-tailed, unpaired *t* test in GraphPad V8). **F** MOLM13-Menin-FKBP12^F36V^ cells were treated with the indicated concentrations of abemaciclib for 96 h without or with 500 nM of dTAG-13. The % of TO-PRO-3 iodide-positive cells was determined by flow cytometry. Mean of two independent experiments performed in duplicate ±S.D. **p < 0.01 compared to MOLM13-Menin-FKBP12^F36V^ cells not treated with dTAG-13 (determined by a two-tailed, unpaired *t* test in GraphPad V8).

We first determined the effects of CRISPR-Cas9-mediated KO of Menin on MLL1/MLL1-FP targets in AML MOLM13 cells that harbor MLL1-r, FLT3-ITD, and two copies of wild-type TP53. Figure 1A demonstrates that 5-days following transfection of Cas9 and two gRNAs targeting exon 2 and 6 of Menin into MOLM13 cells, Menin levels were significantly depleted (Fig. 1A and Fig. S1A), and this was associated with a reduction in MEIS1, FLT3, HOXA9, PBX3, MEF2C, CDK6, p27, and BCL2 levels, but increase in levels of CD11b. We next determined whether the decline in BCL2 and CDK6 levels due to Menin–KO would sensitize MOLM13 cells to the BCL2 inhibitor venetoclax or CDK6 inhibitor abemaciclib. Whereas Menin depletion alone minimally induced loss of viability, it significantly increased sensitivity of MOLM13 cells to venetoclax or abemaciclib-induced loss of viability in a dose-dependent manner (Fig. 1B, C). We also employed the dTAG-13 system to degrade Menin-FKBP12^F36V^ in MOLM13 cells, following near-complete KO of the endogenous Menin, to assess biologic effects in MOLM13 cells ectopically transduced with and expressing Menin-FKBP12^F36V^ [26]. A near-complete KO of the endogenous Menin was achieved by transducing two splice-blocking sgRNAs, one in the intron between exon 3 and exon 4 and the other in the intron between exon 5 and exon 6. As shown in Fig. 1D and Fig. S1B, dTAG-13 treatment for 72 h reduced protein levels of HA-Menin, MEIS1, FLT3, p27, CDK6, and BCL2 in MOLM13 cells. dTAG-13 treatment minimally induced loss of survival of MOLM13 cells (Fig. 1E, F). However, treatment with dTAG-13 significantly increased venetoclax and abemaciclib-induced loss of survival in MOLM13 cells in a dose-dependent manner (Fig. 1E, F).

### Treatment with MI induces differentiation and loss of viability in AML cells harboring MLL1r or NPM1c

**Fig. 2:**
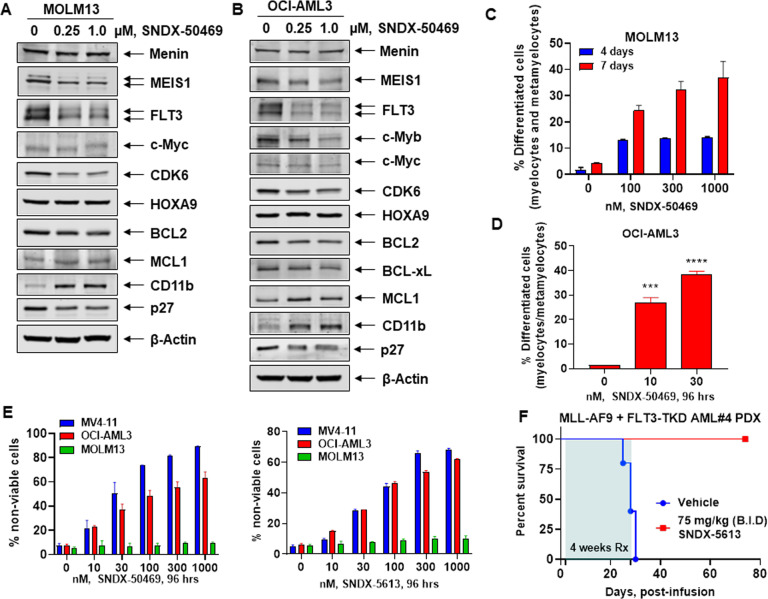
Treatment with Menin inhibitor SNDX-50469 depletes MEIS1, FLT3, CDK6, and BCL2 with concomitant induction of MCL1 and CD11b expression and features of morphologic differentiation in AML cells. **A**, **B** MOLM13 and OCI-AML3 cells were treated with the indicated concentrations of SNDX-50469 for 48 h. Following this, total cell lysates were prepared and immunoblot analyses were conducted. The expression levels of β-Actin in the lysates served as the loading control. **C**, **D** MOLM13 and OCI-AML3 cells were treated with the indicated concentrations of SNDX-50469 for 96 h or 7 days. Morphologic differentiation (% myelocytes, meta-myelocytes, or bands) was determined by light microscopy. Mean of three experiments ±S.E.M. ***p < 0.005; ****p < 0.001 (determined by two-tailed, unpaired *t* test in GraphPad V8). **E** MV4-11, OCI-AML3, and MOLM13 cells were treated with the indicated concentrations of SNDX-50469 or SNDX-5613 for 96 h. At the end of treatment, the % of TO-PRO-3 iodide-positive, non-viable cells were determined by flow cytometry. Columns, mean of three experiments ±S.E.M. **F** Kaplan–Meier survival curve of mice infused with MLL-AF9 + FLT3-TKD expressing PDX (AML#4) cells and treated with SNDX-5613 as indicated for 4 weeks.

We next determined effects of treatment with the pre-clinical MI drug candidate SNDX-50469 (VTP50469) and the closely related clinical drug SNDX-5613 (Syndax Pharmaceuticals) (Fig. S1C) in MOLM13, MV4-11 (harboring MLL1-AF4), and OCI-AML3 cells (expressing NPM1c, homozygous activating NRAS-Q61L and heterozygous DNMT3A-R882C mutations) [27]. Treatment with SNDX-50469 attenuated protein levels of MEIS1, FLT3, p27, CDK6, and BCL2, without affecting Menin, HOXA9, and c-Myc in MOLM13 and OCI-AML3 cells (Figs. 2A, B and S1D). Additionally, SNDX-50469 treatment increased protein levels of MCL1 and BFL1 (gene product of BCL2A1), as well as of PUMA, NOXA and the myeloid differentiation-associated marker CD11b in MOLM13 and OCI-AML3 cells (Figs. 2A, B and S1D, E) [26, 28, 29]. In OCI-AML3 cells, SNDX-50469 treatment also depleted Bcl-xL levels (Fig. 2B). However, SNDX-50469 treatment upregulated BFL1 and MCL1 levels, despite inhibiting p-ERK1/2 but not ERK1/2 levels. This suggested that SNDX-50469 treatment results in inhibition of the MEK kinase previously noted to stabilize MCL1 (Fig. 2B and S1E) [30]. However, co-treatment with the MEK inhibitor cobimetinib not only inhibited p-ERK1/2 levels but also abrogated SNDX-50469-induced MCL1, and modestly inhibited BFL1 levels (Fig. S1F) [31]. This was associated with more apoptosis induced by SNDX-50469 and cobimetinib than treatment with each drug alone (Fig. S1G). This makes it unlikely that MEK inhibition is the cause of SNDX-50469 mediated increase in MCL1 levels (Fig. S1F). Notably, exposure to SNDX-50469 for 4–7 days induced morphologic features of differentiation, as determined by % increase in myelocytes, metamyelocytes, or bands by morphology in hematoxylin & eosin-stained cytospun MOLM13, MV4-11, and OCI-AML3 cells (Figs. 2C, D and S2A, B). Treatment with SNDX-50469 or SNDX-5613 for 4 days dose-dependently also induced loss of viability in MV4-11 and OCI-AML3, but not in MOLM13 cells (Fig. 2E). In MOLM13 cells this was observed only after a 7-day exposure to SNDX-50469 (Fig. S2C). We next determined the effect of SNDX-5613 on in vivo leukemia-initiating potential of primary PD AML cells harboring MLL-AF9 plus FLT-3 N676T, as well as mutations in KMT2C, KMT2D, NOTCH2, IRF8, ARID1A, VPS13A, and ABCA7, which were determined by whole-exome sequencing (Table S1). Figure 2F demonstrates that, following treatment with SNDX-5613, started 24 h after infusion of the PD AML cells into NSG mice, and continued for 4 weeks, all drug-treated mice survived whereas all vehicle-treated mice perished in 4–5 weeks.

### Effect of MI on active chromatin and expression of loci affected by it

**Fig. 3:**
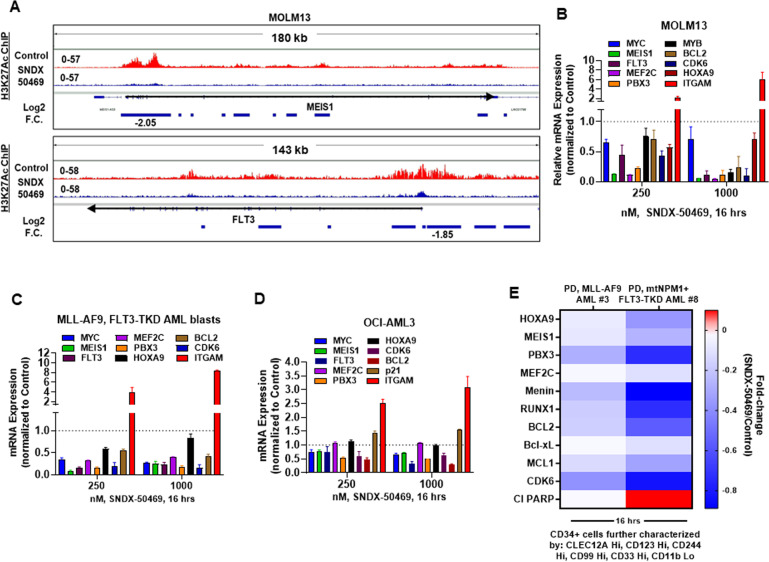
Menin inhibitor treatment depletes H3K27 acetyl mark on chromatin, depletes mRNA expression of MYC, MEIS1, and FLT3 in cultured and primary AML blasts and depletes Menin, BCL2, and MLL target gene expression levels in phenotypically defined leukemia stem cells. **A** IGV plots showing signal tag density of H3K27Ac ChIP-Seq at the MEIS1 and FLT3 gene in MOLM13 cells treated with SNDX-50469 for 16 h. Black arrows mark the direction of the coding sequence of each gene. Blue bars beneath the gene indicate significant, log2 fold-changes in signal tag density of H3K27Ac in SNDX-50469-treated cells compared to control cells. **B**–**D** MOLM13, OCI-AML3, and PD MLL-AF9, FLT3-TKD AML cells were treated with the indicated concentrations of SNDX-50469 for 16 h. Total RNA was isolated, and reverse transcribed. The resulting cDNAs were used for qPCR as shown. The expression of GAPDH served as the normalization control. **E** Patient-derived AML cells were treated with 1000 nM of SNDX-50469 for 16 h. Cells were harvested and analyzed by CyTOF analysis utilizing a cocktail of rare metal element-tagged antibodies. Leukemia stem cells were defined by high expression of CLEC12A, CD123, CD244, CD99, and CD33 but low expression of CD11b.

We next determined whether treatment with SNDX-50469 that results in attenuation of targets of MLL-FP/MLL1 is also associated with repression of the active chromatin and mRNA of these targets in MLL1r AML cells. Utilizing ChIP-Seq analysis, we determined the effect of SNDX-50469 on H3K27Ac signal density at the target loci in MOLM13 cells. As shown in the IGV plots, (Fig. 3A) treatment of MOLM13 cells with SNDX-50469 caused a marked log2-fold reduction in H3K27Ac signal-density at the *cis*-regulatory regions of MEIS1 and FLT3. A similar log2-fold reduction in H3K27Ac peak density was also observed at BCL2, CDK6, and PBX3 loci (Fig. S3). This inhibition of active chromatin at the enhancers and promoters of these loci was associated with inhibition of the mRNA expression of MEIS1, FLT3, PBX3, HOXA9, MYB, MEF2C, MYC, CDK6, and BCL2 but upregulation of the mRNA of ITGAM in MOLM13 cells (Fig. 3B). Similar perturbations in mRNA expressions were also observed in PD AML cells harboring MLL-AF9 and FLT3-TKD mutation and OCI-AML3 cells (Fig. 3C, D). However, in OCI-AML3 cells, SNDX-50469 treatment did not inhibit mRNA expression of HOXA9 and MEF2C mRNA expression (Fig. 3D). Utilizing CyTOF analyses, we next determined the effect of SNDX-50469 treatment on fold-change in protein levels in two samples of phenotypically characterized, PD, CD34 + sorted AML progenitor cells, based on the low expression of CD11b, but high expression of CLEC12A, CD244, CD99, CD123, and CD33. These cells either harbored MLL-AF9 or mtNPM1 and FLT3-TKD mutation [32, 33]. Figure 3E demonstrates that treatment with SNDX-50469 depleted protein expressions of HOXA9, MEIS1, PDX3, MEF2C, Menin, RUNX1, BCL2, Bcl-xL, MCL1, and CDK6. SNDX-50469 treatment increased cleaved PARP levels only in PD AML stem/progenitor cells expressing mtNPM1 and FLT3-TKD.

### The synergistic lethal activity of co-treatment with MI and BCL2 or CDK6 inhibitor in AML cells harboring MLL1r or mtNPM1

**Fig. 4:**
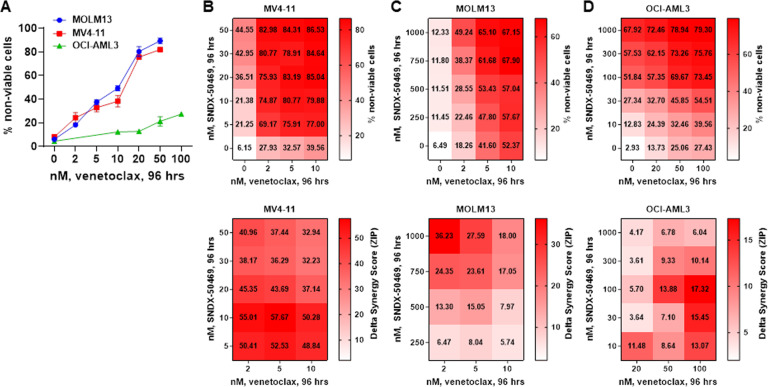
Co-treatment with Menin inhibitor and venetoclax exerts synergistic in vitro lethality in cultured AML cells expressing MLL1-r or mtNPM1. **A** MV4-11, MOLM13, and OCI-AML3 cells were treated with the indicated concentrations of venetoclax for 96 h. At the end of treatment, the % of TO-PRO-3 iodide-positive, non-viable cells were determined by flow cytometry. Columns, mean of three experiments ±S.E.M. **B**–**D**. MV4-11, MOLM13, and OCI-AML3 cells were treated with the indicated concentrations of SNDX-50469 and/or venetoclax for 96 h. At the end of treatment, the % non-viable cells were determined by staining with TO-PRO-3 iodide and flow cytometry analysis. Delta synergy scores were determined by the ZIP method. Synergy scores > 1.0 indicate a synergistic interaction of the two agents in the combination.

**Fig. 5:**
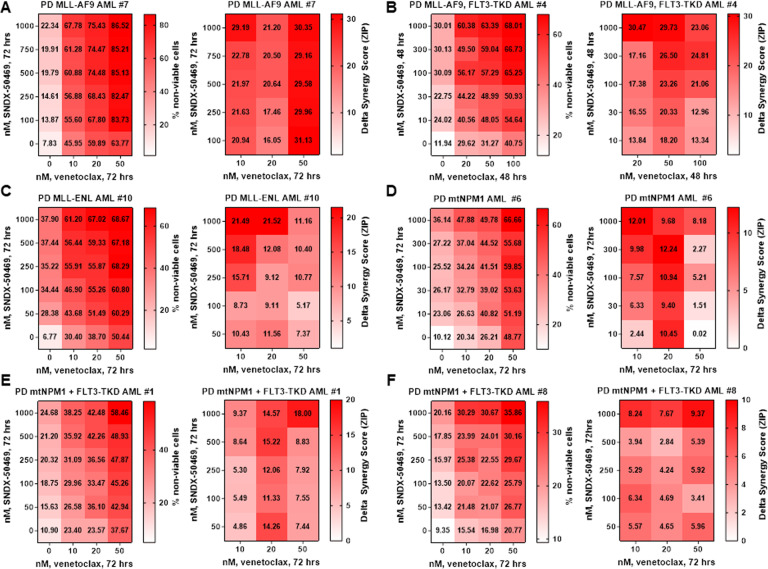
Combined treatment with Menin inhibitor and venetoclax induces synergistic in vitro lethality in patient-derived AML cells expressing MLL1-r or mtNPM1 with or without FLT3 alterations. **A**–**F** Patient-derived AML cells with MLL1 rearrangement or mtNPM1 with or without FLT3 alterations were treated with the indicated concentrations of SNDX-50469 and/or venetoclax for 48–72 h. The % non-viable cells were determined by staining with TO-PRO-3 iodide and flow cytometry analysis. Delta synergy scores were determined by the ZIP method. Synergy scores > 1.0 indicate a synergistic interaction of the two agents in the combination.

Since treatment with SNDX-50469 was associated with inhibition of protein levels of BCL2 in MOLM13 and OCI-AML3 cells, we investigated whether this sets up these cells to be more susceptible to the inhibitory activity of co-treatment with venetoclax on the residual BCL2 levels, as well as increasing anti-AML efficacy of the combination of SNDX-50469 with venetoclax in AML cells expressing MLL-FP or NPM1c. First, we determined that exposure to venetoclax alone dose-dependently induced in vitro loss of viability in MOLM13 and MV4-11 cells (Fig. 4A). However, this effect was relatively muted in OCI-AML3 cells (Fig. 4A). We next determined in vitro synergistic activity of co-treatment with SNDX-50469 and venetoclax against AML cell lines and PD AML cells expressing MLL-FP or NPM1c. Treatment with this combination at relatively low concentrations of each drug exerted high levels of synergistic lethality against MV4-11, MOLM13, and OCI-AML3 cells, with delta synergy scores greater than five (Fig. 4B–D). This synergy, due to the combination, was observed despite relatively lower level of lethal activity of either SNDX-50469 or venetoclax alone in MOLM13 and OCI-AML3 cells, respectively (Fig. 4C, D). Additionally, co-treatment with SNDX-5613 and venetoclax was also synergistically lethal against MOLM13 and OCI-AML3 cells (Fig. S4A, B). Thus, by lowering both the levels and antiapoptotic activity of BCL2, co-treatment with MI and venetoclax is more effective in lowering the threshold and augmenting apoptosis, as compared to treatment with each drug alone. Since SNDX-50469 treatment also inhibited CDK6 levels, we determined the lethal activity of co-treatment with SNDX-50469 and abemaciclib (CDK6 inhibitor) [34, 35]. Indeed, a combination of SNDX-50469 and abemaciclib was also synergistically lethal against MOLM13 and MV4-11 cells, but only additive against OCI-AML3 cells (Fig. S4C, D, and data not shown). We conducted additional studies to interrogate the effects of SNDX-50469 and abemaciclib on induction of senescence in MOLM13 and OCI-AML3 cells. We evaluated this by estimating the effects of SNDX-50469 and/or abemaciclib on the markers of senescence, i.e., β-galactosidase and p16 levels, as well as on levels of pRB and RB proteins [36]. As shown in Figure S4E, whereas treatment with abemaciclib significantly induced β-galactosidase levels in MOLM13 and OCI-AML3 cells, SNDX-50469 treatment did not. Co-treatment with SNDX-50469 did not augment β-galactosidase induced by abemaciclib treatment (Fig. S4E). SNDX-50469 and/or abemaciclib did not affect levels of p16, another marker of senescence, in MOLM13 and OCI-AML3 cells (Fig. S4F, G). Similar to the synergy observed in cultured cell lines, co-treatment with SNDX-50469 and venetoclax also displayed synergistic lethal activity against PD AML cells harboring MLL-AF9 (sample #7), MLL-AF9 plus FLT3-TKD (sample #4), MLL-ENL (sample #10), mtNPM1 (sample #6), mtNPM1 plus FLT3-TKD (samples #1, #5, and #8), and mtNPM1 plus FLT3-ITD and FLT3-TKD (sample #11) (Figs. 5A–F and S5A–C). Additionally, the combination of SNDX-50469 and abemaciclib was also synergistically lethal against PD AML cells harboring MLL-AF9 or mtNPM1 plus mutant FLT3 (samples #4, #5, and #9) (Fig. S5D–F). In contrast, Fig. S5G demonstrates that treatment with SNDX-50469 and/or venetoclax or abemaciclib only minimally increased in vitro loss of viability in normal CD34+ progenitor cells, which remained below 15% following exposure to SNDX-50469 or its combination with venetoclax or abemaciclib.

### MI-based combinations retain lethal activity against MLL1-r AMLs harboring genetic lesions in TP53

**Fig. 6:**
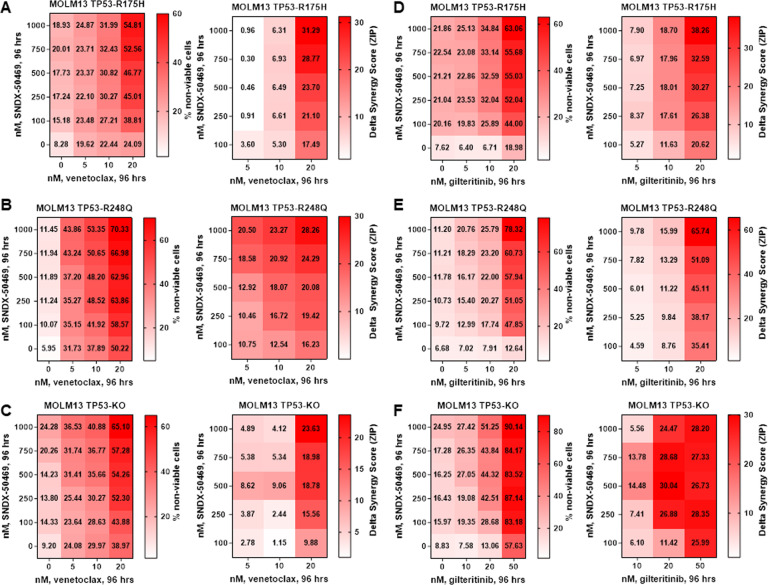
Co-treatment with Menin inhibitor and venetoclax or FLT3 inhibitor gilteritinib induces synergistic in vitro lethality in MOLM13 cells with isogenic TP53 mutations or TP53 knockout. **A**–**C** MOLM13 TP53-R175H, MOLM13 TP53-R248Q, and MOLM13 TP53^−/−^ cells were treated with the indicated concentrations of SNDX-50469 and/or venetoclax for 96 h. At the end of treatment, the % non-viable cells were determined by staining with TO-PRO-3 iodide and flow cytometry analysis. Delta synergy scores were calculated by the ZIP method. Synergy scores > 1.0 indicate a synergistic interaction of the two agents in the combination. **D**–**F** MOLM13 TP53-R175H, MOLM13 TP53-R248Q, and MOLM13 TP53^−/−^ cells were treated with the indicated concentrations of SNDX-50469 and/or gilteritinib for 96 h. At the end of treatment, the % non-viable cells were determined by staining with TO-PRO-3 iodide and flow cytometry analysis. Delta synergy scores were determined by the ZIP method. Synergy scores > 1.0 indicate a synergistic interaction of the two agents in the combination.

From 2012 to 2021, 190 patients with the diagnosis of AML with MLL1-r and genetic lesion(s) in TP53 were managed in the Leukemia Department of MD Anderson Cancer Center. Of these, 17 patients (~9%) harbored mutations in TP53 (Table S2). These included commonly observed, hotspot, missense mutations in the DNA binding domain of TP53, such as R175H and R248Q [37]. Utilizing CRISPR/Cas9, these missense mutations, or null alleles, were introduced into the endogenous locus of MOLM13 cells that carry two wild-type copies of TP53, as previously reported [38]. These isogenic cells lines enable comparison of mutant, wild-type, and null alleles of TP53 expressed from the endogenous locus. For example, MOLM13 harboring missense mutation of TP53 (MOLM13-mtTP53 cells) were significantly less sensitive to the DNA-damaging drug etoposide than MOLM13 (Fig. S6A, and not shown). In contrast, MOLM13-mtTP53 cells were as sensitive as MOLM13 cells to SNDX-50469-induced morphologic differentiation and loss of viability (Fig. S6B–D). We next determined the effect of SNDX-50469 on mRNA and protein expression of MLL1-r targets in MOLM13 TP53-R175H and TP53-R248Q cells. Figure S6E demonstrates that exposure to SNDX-50469 was associated with repression of MEIS1, PBX3, MEF2C, MYC, FLT3, CDK6, and BCL2, but upregulation of ITGAM mRNA levels. It was also associated with attenuated protein expression of MEIS1, FLT3, CDK6, BCL2, and p27, but upregulation CD11b (Fig. S6F). Based on these findings, we then determined the lethal activity of SNDX-50469 alone and of co-treatment with SNDX-50469 and venetoclax or gilteritinib, a type I FLT3 kinase inhibitor [39], on MOLM13 TP53-R175H, TP53-R248Q, and TP53^−/−^ cells. Although displaying a low level of activity when SNDX-50469 was administered alone, co-treatment with SNDX-50469 and venetoclax induced synergistic loss of viability in MOLM13 TP53-R175H, TP53-R248Q, and TP53^−/−^ cells displaying delta synergy scores above 2.5 by the ZIP method (Fig. 6A–C). These results highlight that the synergy of the combination was not lessened by introduction of the genetic lesions of TP53 in MOLM13 cells. Since MOLM13 cells also harbor an FLT3-ITD mutation, we determined the synergistic lethal activity of co-treatment with SNDX-50469 and gilteritinib against MOLM13, as well as against MOLM13 TP53-R175H, TP53-R248Q, and TP53^−/−^ cells [40]. As shown in Fig. 6D–F, gilteritinib alone exerted modest lethal activity in MOLM13 and MOLM13 TP53-R175H or TP53-R248Q, but higher loss of viability in TP53^−/−^ cells. However, co-treatment with SNDX-50469 and gilteritinib induced synergistic loss of survival in MOLM13, MOLM13 TP53-R175H, TP53-R248Q, and TP53^−/−^ cells, displaying delta synergy scores above 4.5 by the ZIP method (Figs. 6D–F and S6G). This again highlights that the synergy of this combination was also not compromised by introducing the genetic lesions of TP53 in MOLM13 cells. Co-treatment with SNDX-50469 and gilteritinib also synergistically induced loss of viability in PD AML cells harboring mtNPM1 and FLT3-ITD and FLT3-F691L mutations (Fig. S6H).

### Superior in vivo efficacy of the combination of MI with venetoclax against AML cells harboring MLL1-r or mtNPM1

**Fig. 7:**
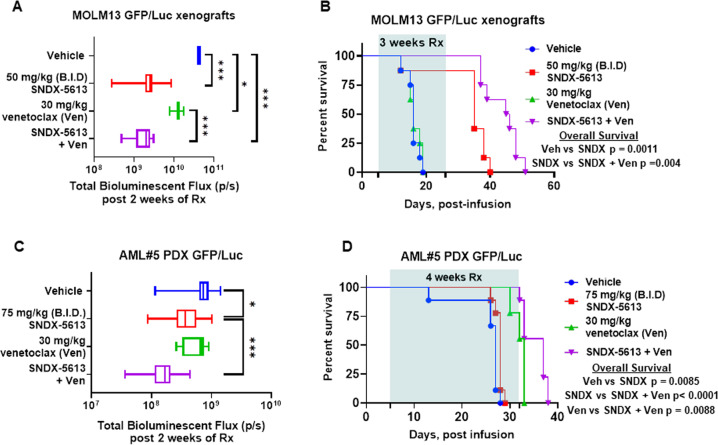
Treatment with SNDX-5613 and venetoclax reduced leukemia burden and significantly improved survival of NSG mice bearing MLL1-r or mtNPM1 with mtFLT3 AML xenografts. **A** Total photon count [flux] (determined by bioluminescent imaging) in NSG mice engrafted with MOLM13 GFP/Luc cells and treated for 2 weeks with SNDX-5613 and/or venetoclax at the indicated doses. **B** Kaplan–Meier survival plot of NSG mice engrafted with MOLM13 GFP/Luc cells and treated with 50 mg/kg of SNDX-5613 (B.I.D. ×5 days, P.O.) and/or 30 mg/kg of venetoclax (daily ×5 days, P.O.) for 3 weeks. Significance was calculated by a Mantel–Cox log-rank test. **C** Total photon counts [flux] (determined by bioluminescent imaging) in NSG mice engrafted with PD, AML blasts expressing mtNPM1, and mtFLT3 and treated with vehicle or SNDX-5613 and/or venetoclax at the indicated doses for 2 weeks. **D** Kaplan–Meier survival plot of NSG mice bearing a mtNPM1 and mtFLT3-expressing AML PDX and treated with 75 mg/kg of SNDX-5613 (B.I.D. ×5 days, P.O.) and/or 30 mg/kg of venetoclax (daily ×5 days, P.O.) for 4 weeks. Significance was calculated by a Mantel–Cox log-rank test.

Given the marked in vitro synergy of SNDX-50469 or SNDX-5613 combination with venetoclax against AML cells harboring MLL1-r or mtNPM1, we next determined in vivo anti-leukemia efficacy of SNDX-5613 and/or venetoclax at well-tolerated dose levels or their combination in NSG mice engrafted with MOLM13-GFP/Luciferase (Luc) cells [41]. Compared to vehicle control, or treatment with venetoclax alone, co-treatment with SNDX-5613 and venetoclax for just two weeks induced a greater reduction in AML burden (Fig. 7A). Whereas treatment with a relatively low dose of venetoclax alone for three weeks was ineffective, SNDX-5613 treatment alone significantly improved median and overall survival of NSG mice engrafted with MOLM13-GFP/Luc cells (*P* < 0.005) (Fig. 7B). The dose of each drug employed here was relatively low and previously determined to be safe [17, 20, 41]. Next, we determined in vivo efficacy of treatment with SNDX-5613 and/or venetoclax against a PDX model in NSG mice engrafted with AML cells harboring mtNPM1, DNMT3A, mtFLT3, IDH1, WT1, KMT2C, as well as other genes listed in Table S3. These cells had been transduced with and expressed GFP/Luciferase for bioluminescence imaging. Again, although monotherapy with SNDX-5613 or venetoclax for just 2 weeks reduced the AML burden, co-treatment with SNDX-5613 and venetoclax reduced AML burden significantly more than treatment with vehicle or with each agent alone (Fig. 7C). Notably, whereas treatment with SNDX-5613 or venetoclax alone for four weeks was effective in improving median and overall survival, co-treatment with SNDX-5613 and venetoclax displayed more efficacy and significantly improved median and overall survival, as compared to treatment with each agent alone (*p* < 0.01) (Fig. 7D). Neither monotherapy with the agents nor treatment with the combinations was associated with weight loss or other toxicities, as compared to mice treated with vehicle alone. These findings indicate that combined therapy with SNDX-5613 and venetoclax is effective as in vivo therapy for AML harboring mutations in multiple genes, including mtNPM1, DNMT3A, IDH1, and mtFLT3, a set of co-mutations most commonly observed in AML with normal karyotype but known to be associated with poor prognosis.

## Discussion

Findings presented here demonstrate for the first time that, by either utilizing the dTAG system to degrade Menin or CRISPR-Cas9 to edit and KO Menin, depletion of Menin is associated with a reduction in expression of MEIS1, FLT3, CDK6, and BCL2, sensitizing MOLM13 cells harboring MLL1-r and FLT3-ITD to BCL2 inhibitor venetoclax and CDK6 inhibitor abemaciclib-induced apoptosis. Five days after transduction of gRNA to Menin, protein levels of HOXA9, MEF2C, PBX3, and p27 were also notably reduced in MOLM13 cells. A previous report had documented that loss of occupancy of Menin, MLL1 (N-terminus), DOT1L, and H3K79Me2 from selective chromatin targets induced concordant repression of a subset of MLL1/MLL-FP target genes [17]. Utilizing ChIP-Seq analysis of H3K27Ac binding, findings presented here demonstrate that MI treatment reduced H3K27Ac occupancy at the active regulatory chromatin of Menin-MLL1 targets MEIS1, FLT3, PBX3, CDK6, and BCL2 [17, 20], with concordant downregulation of their mRNA and/or protein expressions. In contrast, MI treatment upregulated MCL1 and BFL1 protein levels, which were abrogated by co-treatment with MEK inhibitor cobimetinib [31], yielding significantly more apoptosis due to the combination than due to each drug alone. SNDX-50469 treatment reduced p27 expression since Menin was previously reported to transcriptionally upregulate p27 expression in AML cells expressing MLL-FP or mtNPM1 [42]. It is notable that although it did not affect HOXA9 protein levels, SNDX-50469 treatment is likely to have undermined the transcriptional activity of HOXA9 by downregulating the protein levels of its co-factor MEIS1 [20]. Notably, disruption of Menin-MLL1 interaction was previously shown to trigger Menin protein degradation through the ubiquitin–proteasome mechanism [43]. However, at the dose and exposure interval of SNDX-50469 utilized in present studies, intracellular depletion of Menin was not observed. Importantly, consistent with previous reports, our findings also confirm that treatment with MI SNDX-50469 induces differentiation, with upregulation of CD11b expression, associated with loss of viability of AML cells harboring MLL1-r and mtNPM1 [17, 26]. Consistent with reported effects of DOT1L or DHODH (dihydroorotate dehydrogenase) [44], these findings indicate that MI treatment also overcomes differentiation blockage in AML cells with MLL1-r [17, 44]. Previous reports had highlighted that protein-protein interaction between Menin and MLL1 plays a central role in the pathogenesis of MLL1-r AML, and disruption of this interaction by MI treatment blocked the development of AML by cell lines harboring MLL1-r [45]. Findings here confirm this by demonstrating that treatment with the MI SNDX-5613 abrogated the development of AML in a PDX model of AML harboring MLL1-r and mtFLT3. Consistent with previous reports, our findings also demonstrate that MI treatment improves median and overall survival of mice with established AML due to MOLM13 cells [46].

Present studies also show that MI-mediated reduction in expression of protein levels of BCL2 and CDK6 could be therapeutically exploited. They demonstrate that co-treatment with SNDX-50469 and venetoclax or abemaciclib induced synergistic lethality in not only AML cell lines but also PD AML cells that harbor either MLL1-r or mtNPM1c along with FLT3-ITD and/or mtFLT3 (ITD or TKD). These findings were associated with SNDX-50469-mediated downregulation of not only BCL2 and CDK6, as noted above, but also of MCL1 and to a lesser extent Bcl-xL in PD, CD34+ phenotypically characterized (by CyTOF analysis) AML stem/progenitor cells. These perturbations might further explain the synergistic interaction between SNDX-50469 and venetoclax, since overexpression of MCL1 and Bcl-xL have been documented as important mechanisms dampening the pro-apoptotic activity of venetoclax [47]. A recent report documented that Menin inhibition decreases BCL2 and synergizes with venetoclax against NPM1/FLT3 mutated AML [48]. However, our findings here go beyond this and show that MI and venetoclax are synergistically lethal against AML with MLL1-r or mtNPM1 with or without co-expression of FLT3-ITD or FLT3-TKD. A possible explanation for this may be that SNDX-50469 treatment lowers the apoptosis threshold by concomitantly reducing levels of BCL2 and Bcl-xL. Since OCI-AML3 cells also contain a homozygous, gain of function mutation, NRAS-Q61L, our findings also suggest that a combination of MI and venetoclax or abemaciclib may also have superior activity against NPM1/RAS-mutated AML cells [27].

Although observed in more than 50% of epithelial cancers, somatic missense mutations and/or allelic loss of TP53 is sub-clonal and occurs in approximately 10% of patients with MLL1-r AML, and even less common in AML with mtNPM1 [24, 25, 37]. The presence of this TP53 lesion is associated with resistance to apoptosis induced by DNA-damaging AML chemotherapy and relatively poor clinical outcomes [24, 25, 49]. Although treatment with hypomethylating agents with or without venetoclax are currently under investigation, none of these agents have overcome therapy resistance conferred by TP53 lesions and improved clinical outcomes [50, 51]. A previous report had described MOLM13 cells into which TP53 mutations R175H and R248Q had been introduced along with null alleles [38]. Resulting isogenic MOLM13-TP53 cells with either the missense or null alleles faithfully recapitulated p53 biology and showed the same oncogenic phenotype with respect to the proliferative capacity, decreased apoptotic potential, lack of G1 cell cycle arrest, and resistance to cytotoxic agents compared to MOLM13-TP53^+/+^ cells [38]. However, present findings demonstrate that MOLM13 TP53-R175H or -R248Q cells or MOLM13-TP53^−/−^ cells are almost as sensitive as MOLM13-TP53^+/+^ cells to MI-induced loss of viability. Additionally, co-treatment with MI and venetoclax is synergistically lethal against MOLM13 TP53-R175H or -R248Q cells or against MOLM13-TP53^−/−^ cells. Furthermore, consistent with a previous report, the combination of MI with the FLT3 kinase inhibitor gilteritinib exerted synergistic lethality against MOLM13-TP53^+/+^ as well as MOLM13 TP53-R175H or -R248Q cells or MOLM13-TP53^−/−^ cells [39, 40]. Overall, the findings presented here support the promise of further developing and testing of these MI-based combinations in AML with MLL1-r or mtNPM1 with mutations in TP53 and/or FLT3.

## Supplementary information

Supplemental Figures and Tables

Supplemental Figure Legends

Supplemental Materials and Methods

## References

[1] Yu BD, Hanson RD, Hess JL, Horning SE, Korsmeyer SJ (1998). MLL, a mammalian trithorax-group gene, functions as a transcriptional maintenance factor in morphogenesis. Proc Natl Acad Sci USA.

[2] Li X, Song Y (2021). Structure, function and inhibition of critical protein-protein interactions involving mixed lineage leukemia 1 and its fusion oncoproteins. J Hematol Oncol.

[3] Matkar S, Thiel A, Hua X (2013). Menin: a scaffold protein that controls gene expression and cell signaling. Trends Biochem Sci.

[4] Murai MJ, Chruszcz M, Reddy G, Grembecka J, Cierpicki T (2011). Crystal structure of Menin reveals binding site for mixed lineage leukemia (MLL) protein. J Biol Chem.

[5] Yokoyama A, Cleary ML (2008). Menin critically links MLL proteins with LEDGF on cancer-associated target genes. Cancer Cell.

[6] Yokoyama A, Somervaille TC, Smith KS, Rozenblatt-Rosen O, Meyerson M, Cleary ML (2005). The Menin tumor suppressor protein is an essential oncogenic cofactor for MLL-associated leukemogenesis. Cell.

[7] Milne TA, Kim J, Wang GG, Stadler SC, Basrur V, Whitcomb SJ (2010). Multiple interactions recruit MLL1 and MLL1 fusion proteins to the HOXA9 locus in leukemogenesis. Mol Cell.

[8] Argiropoulos B, Yung E, Humphries RK (2007). Unraveling the crucial roles of Meis1 in leukemogenesis and normal hematopoiesis. Genes Dev.

[9] Sun Y, Zhou B, Mao F, Xu J, Miao H, Zou Z (2018). HOXA9 reprograms the enhancer landscape to promote leukemogenesis. Cancer Cell.

[10] McMahon KA, Hiew SY, Hadjur S, Veiga-Fernandes H, Menzel U, Price AJ (2007). Mll has a critical role in fetal and adult hematopoietic stem cell self-renewal. Cell Stem Cell.

[11] Abramovich C, Humphries RK (2005). Hox regulation of normal and leukemic hematopoietic stem cells. Curr Opin Hematol.

[12] Krivtsov AV, Armstrong SA (2007). MLL translocations, histone modifications and leukaemia stem-cell development. Nat Rev Cancer.

[13] Collins CT, Hess JL (2016). Deregulation of the HOXA9/MEIS1 axis in acute leukemia. Curr Opin Hematol.

[14] Yokoyama A, Lin M, Naresh A, Kitabayashi I, Cleary ML (2010). A higher-order complex containing AF4 and ENL family proteins with P-TEFb facilitates oncogenic and physiologic MLL-dependent transcription. Cancer Cell.

[15] Muntean AG, Tan J, Sitwala K, Huang Y, Bronstein J, Connelly JA (2010). The PAF complex synergizes with MLL fusion proteins at HOX loci to promote leukemogenesis. Cancer Cell.

[16] Chen Y, Jones KL, Anastassiadis K, Kranz A, Stewart AF, Grembecka J (2019). Distinct pathways affected by Menin versus MLL1/MLL2 in MLL-rearranged acute myeloid leukemia. Exp Hematol.

[17] Krivtsov AV, Evans K, Gadrey JY, Eschle BK, Hatton C, Uckelmann HJ (2019). A Menin-MLL Inhibitor Induces Specific Chromatin Changes and Eradicates Disease in Models of MLL-Rearranged Leukemia. Cancer Cell.

[18] Falini B, Brunetti L, Sportoletti P, Martelli MP (2020). NPM1-mutated acute myeloid leukemia: from bench to bedside. Blood.

[19] Brunetti L, Gundry MC, Sorcini D, Guzman AG, Huang YH, Ramabadran R (2018). Mutant NPM1 maintains the leukemic state through HOX expression. Cancer Cell.

[20] Uckelmann HJ, Kim SM, Wong EM, Hatton C, Giovinazzo H, Gadrey JY (2020). Therapeutic targeting of preleukemia cells in a mouse model of NPM1 mutant acute myeloid leukemia. Science.

[21] Borkin D, He S, Miao H, Kempinska K, Pollock J, Chase J (2015). Pharmacologic inhibition of the Menin-MLL interaction blocks progression of MLL leukemia in vivo. Cancer Cell.

[22] Klossowski S, Miao H, Kempinska K, Wu T, Purohit T, Kim E (2020). Menin inhibitor MI-3454 induces remission in MLL1-rearranged and NPM1-mutated models of leukemia. J Clin Invest.

[23] Issa GC, Ravandi F, DiNardo CD, Jabbour E, Kantarjian HM, Andreeff M (2021). Therapeutic implications of Menin inhibition in acute leukemias. Leukemia.

[24] Prokocimer M, Molchadsky A, Rotter V (2017). Dysfunctional diversity of p53 proteins in adult acute myeloid leukemia: projections on diagnostic workup and therapy. Blood.

[25] Short NJ, Montalban-Bravo G, Hwang H, Ning J, Franquiz MJ, Kanagal-Shamanna R (2020). Prognostic and therapeutic impacts of mutant TP53 variant allelic frequency in newly diagnosed acute myeloid leukemia. Blood Adv.

[26] Fiskus W, Mill CP, Nabet B, Perera D, Birdwell C, Manshouri T (2021). Superior efficacy of co-targeting GFI1/KDM1A and BRD4 against AML and post-MPN secondary AML cells. Blood Cancer J.

[27] Simonsen AT, Hansen MC, Kjeldsen E, Moller PL, Hindkjaer JJ, Hokland P (2018). Systematic evaluation of signal-to-noise ratio in variant detection from single cell genome multiple displacement amplification and exome sequencing. BMC Genomics.

[28] Hernandez Borrero LJ, El-Deiry WS (2021). Tumor suppressor p53: Biology, signaling pathways, and therapeutic targeting. Biochim. Biophys. Rev. Cancer.

[29] Ashkenazi A, Fairbrother WJ, Leverson JD, Souers AJ (2017). From basic apoptosis discoveries to advanced selective BCL-2 family inhibitors. Nat Rev Drug Discov.

[30] Han L, Zhang Q, Dail M, Shi C, Cavazos A, Ruvolo VR (2020). Concomitant targeting of BCL2 with venetoclax and MAPK signaling with cobimetinib in acute myeloid leukemia models. Haematologica.

[31] Seipel K, Marques MAT, Sidler C, Mueller BU, Pabst T (2018). The cellular p53 inhibitor MDM2 and the growth factor receptor FLT3 as biomarkers for treatment responses to the MDM2-inhibitor idasanutlin and the MEK1 inhibitor cobimetinib in acute myeloid leukemia. Cancers (Basel).

[32] Behbehani GK, Samusik N, Bjornson ZB, Fantl WJ, Medeiros BC, Nolan GP (2015). Mass cytometric functional profiling of acute myeloid leukemia defines cell-cycle and immunophenotypic properties that correlate with known responses to therapy. Cancer Discov.

[33] Saenz DT, Fiskus W, Manshouri T, Mill CP, Qian Y, Raina K (2019). Targeting nuclear beta-catenin as therapy for post-myeloproliferative neoplasm secondary AML. Leukemia.

[34] Dowless M, Lowery CD, Shackleford T, Renschler M, Stephens J, Flack R (2018). Abemaciclib is active in preclinical models of Ewing sarcoma via multipronged regulation of cell cycle, DNA methylation, and interferon pathway signaling. Clin Cancer Res.

[35] Torres-Guzman R, Calsina B, Hermoso A, Baquero C, Alvarez B, Amat J (2017). Preclinical characterization of abemaciclib in hormone receptor positive breast cancer. Oncotarget.

[36] Gorgoulis V, Adams PD, Alimonti A, Bennett DC, Bischof O, Bishop C (2019). Cellular senescence: defining a path forward. Cell.

[37] Kastenhuber ER, Lowe SW (2017). Putting p53 in context. Cell.

[38] Boettcher S, Miller PG, Sharma R, McConkey M, Leventhal M, Krivtsov AV (2019). A dominant-negative effect drives selection of TP53 missense mutations in myeloid malignancies. Science.

[39] Levis M, Perl AE (2020). Gilteritinib: potent targeting of FLT3 mutations in AML. Blood Adv.

[40] Dzama MM, Steiner M, Rausch J, Sasca D, Schonfeld J, Kunz K (2020). Synergistic targeting of FLT3 mutations in AML via combined Menin-MLL and FLT3 inhibition. Blood.

[41] Fiskus W, Cai T, DiNardo CD, Kornblau SM, Borthakur G, Kadia TM (2019). Superior efficacy of cotreatment with BET protein inhibitor and BCL2 or MCL1 inhibitor against AML blast progenitor cells. Blood Cancer J.

[42] Milne TA, Hughes CM, Lloyd R, Yang Z, Rozenblatt-Rosen O, Dou Y (2005). Menin and MLL cooperatively regulate expression of cyclin-dependent kinase inhibitors. Proc Natl Acad Sci USA.

[43] Wu Y, Doepner M, Hojnacki T, Feng Z, Katona BW, He X (2019). Disruption of the Menin-MLL interaction triggers Menin protein degradation via ubiquitin-proteasome pathway. Am J Cancer Res.

[44] Dafflon C, Craig VJ, Mereau H, Grasel J, Schacher Engstler B, Hoffman G (2017). Complementary activities of DOT1L and Menin inhibitors in MLL-rearranged leukemia. Leukemia.

[45] Borkin D, Klossowski S, Pollock J, Miao H, Linhares BM, Kempinska K (2018). Complexity of blocking bivalent protein-protein interactions: development of a highly potent inhibitor of the Menin-mixed-lineage leukemia interaction. J Med Chem.

[46] Miao H, Kim E, Chen D, Purohit T, Kempinska K, Ropa J (2020). Combinatorial treatment with Menin and FLT3 inhibitors induces complete remission in AML models with activating FLT3 mutations. Blood.

[47] Yue X, Chen Q, He J (2020). Combination strategies to overcome resistance to the BCL2 inhibitor venetoclax in hematologic malignancies. Cancer Cell Int.

[48] Carter BZ, Tao W, Mak PY, Ostermann LB, Mak DH, McGeehan GM (2021). Menin inhibition decreases Bcl-2 and synergizes with venetoclax in NPM1/FLT3-mutated AML. Blood.

[49] Yan B, Claxton D, Huang S, Qiu Y (2020). AML chemoresistance: the role of mutant TP53 subclonal expansion and therapy strategy. Exp Hematol.

[50] Kim K, Maiti A, Loghavi S, Pourebrahim R, Kadia TM, Rausch CR (2021). Outcomes of TP53-mutant acute myeloid leukemia with decitabine and venetoclax. Cancer.

[51] Venugopal S, Shoukier M, Konopleva M, Dinardo CD, Ravandi F, Short NJ (2021). Outcomes in patients with newly diagnosed TP53-mutated acute myeloid leukemia with or without venetoclax-based therapy. Cancer.

