# Supplementary material for: Effective Menin inhibitor-based combinations against AML with MLL rearrangement or NPM1 mutation (NPM1c)
Source: Blood Cancer J. 2022 Jan 11;12(1):5. doi: 10.1038/s41408-021-00603-3 (PMC8752621; doi:10.1038/s41408-021-00603-3)
Supplement: Supplementary file 1 — Supplemental Figures and Tables [file 41408_2021_603_MOESM1_ESM.pdf]

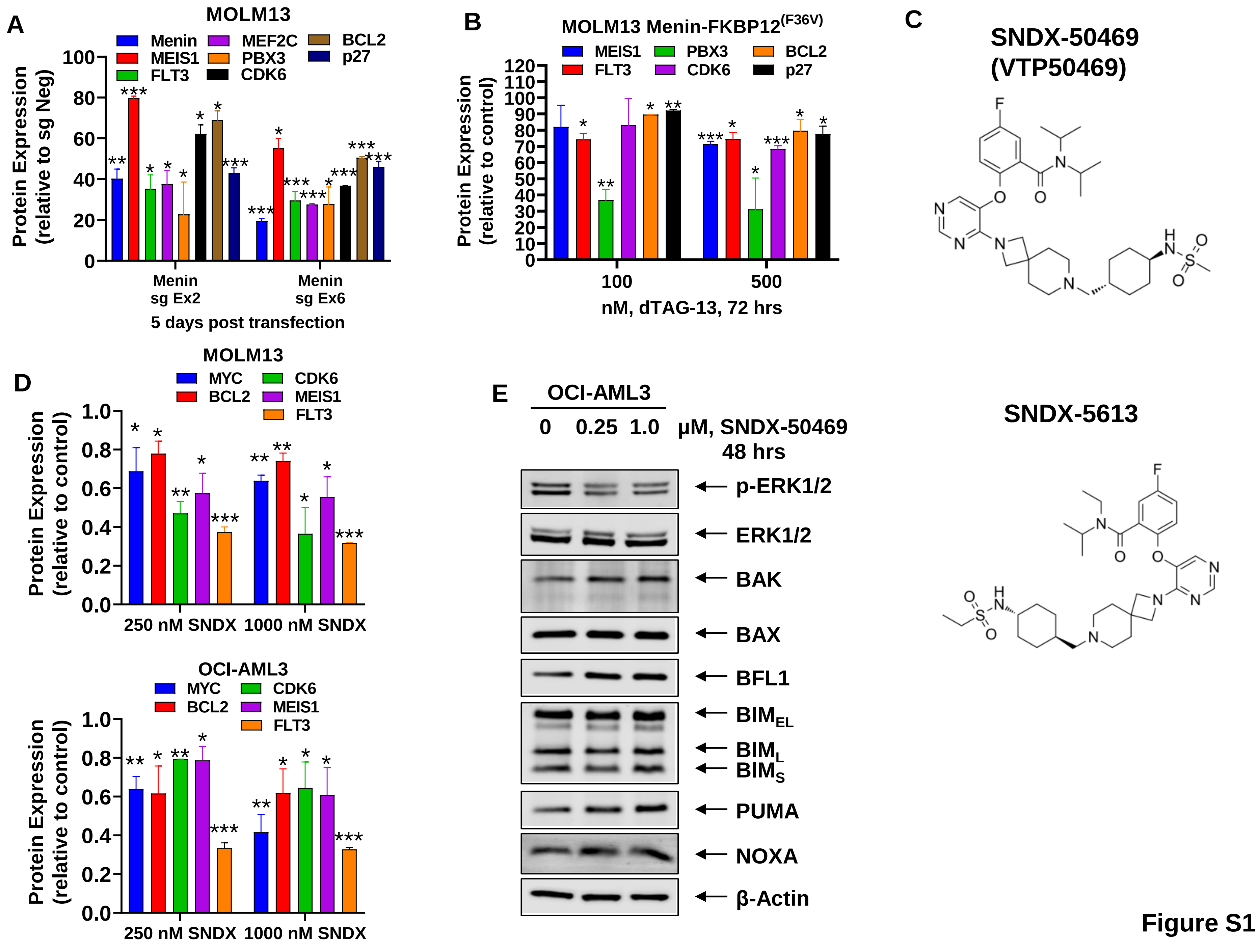
A
MOLM13
Menin
MEIS1
FLT3
MEF2C
PBX3
CDK6
BCL2
p27
Protein Expression (relative to sg Neg)
Menin sg Ex2
Menin sg Ex6
5 days post transfection
B
MOLM13 Menin-FKBP12(F36V)
MEIS1
FLT3
PBX3
CDK6
BCL2
p27
Protein Expression (relative to control)
100
500
nM, dTAG-13, 72 hrs
C
SNDX-50469 (VTP50469)
SNDX-5613
D
MOLM13
MYC
BCL2
CDK6
MEIS1
FLT3
Protein Expression (relative to control)
250 nM SNDX
1000 nM SNDX
OCI-AML3
E
OCI-AML3
0
0.25
1.0
μM, SNDX-50469 48 hrs
p-ERK1/2
ERK1/2
BAK
BAX
BFL1
BIMEL
BIML
BIMS
PUMA
NOXA
β-Actin

Figure S1

F

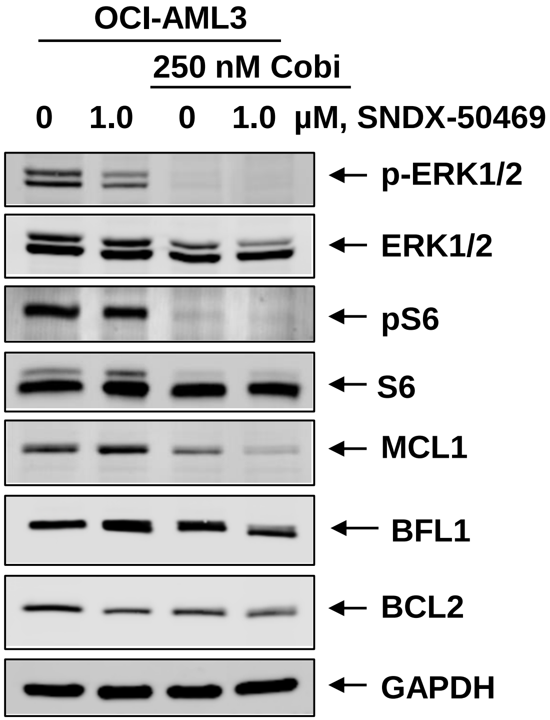
OCI-AML3
250 nM Cobi
0 1.0 0 1.0 μM, SNDX-50469
p-ERK1/2
ERK1/2
pS6
S6
MCL1
BFL1
BCL2
GAPDH

G

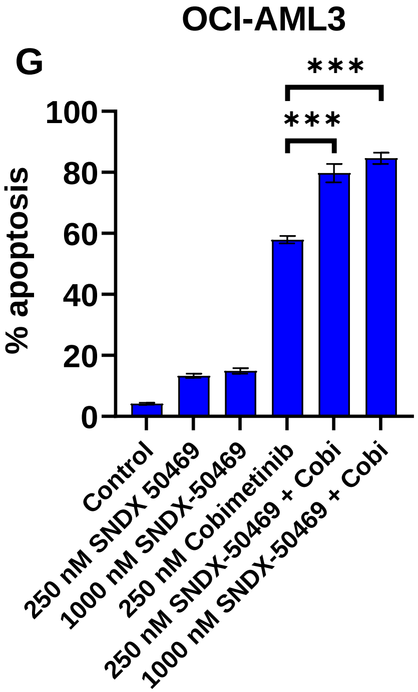
OCI-AML3
***
***
% apoptosis
0
20
40
60
80
100
Control
250 nM SNDX 50469
1000 nM SNDX-50469
250 nM Cobimetinib
250 nM SNDX-50469 + Cobi
1000 nM SNDX-50469 + Cobi

Figure S1

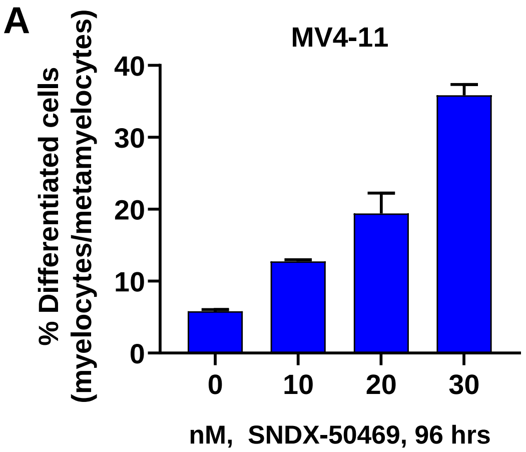
A
MV4-11
% Differentiated cells (myelocytes/metamyelocytes)
0
10
20
30
40
0
10
20
30
nM, SNDX-50469, 96 hrs

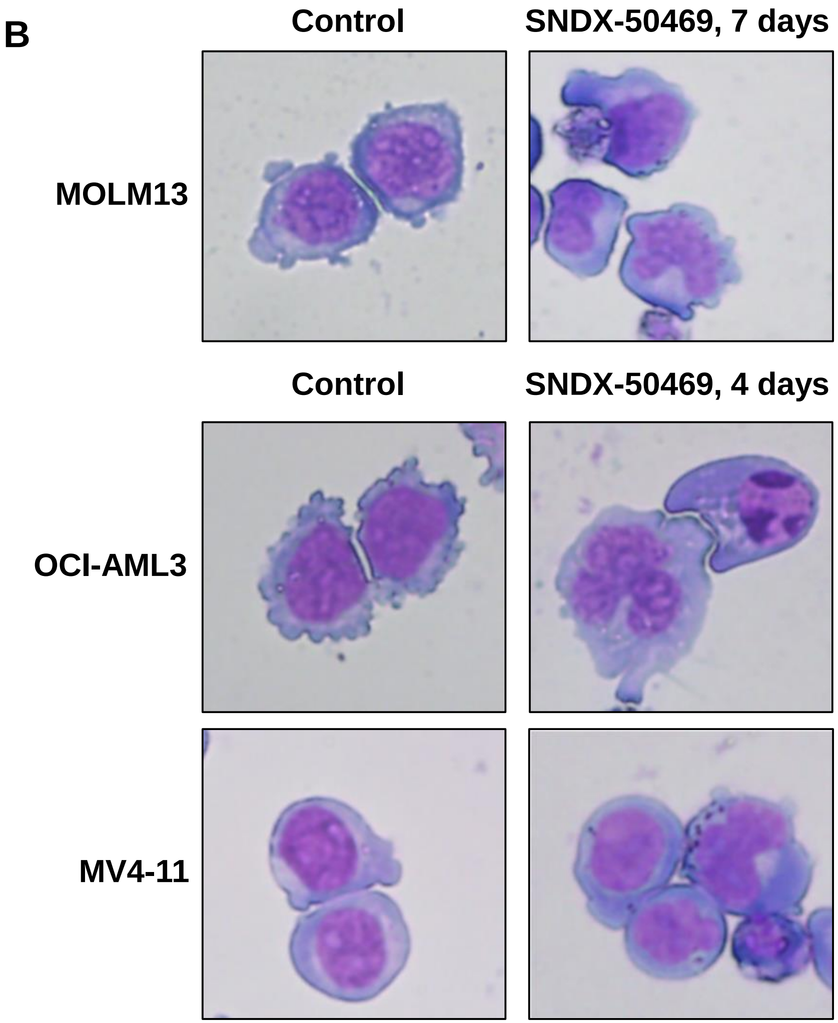
B
Control
SNDX-50469, 7 days
MOLM13
Control
SNDX-50469, 4 days
OCI-AML3
MV4-11

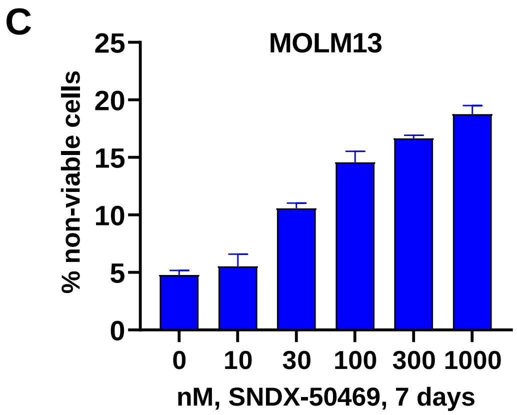
C
MOLM13
% non-viable cells
0
5
10
15
20
25
0
10
30
100
300
1000
nM, SNDX-50469, 7 days

Figure S2

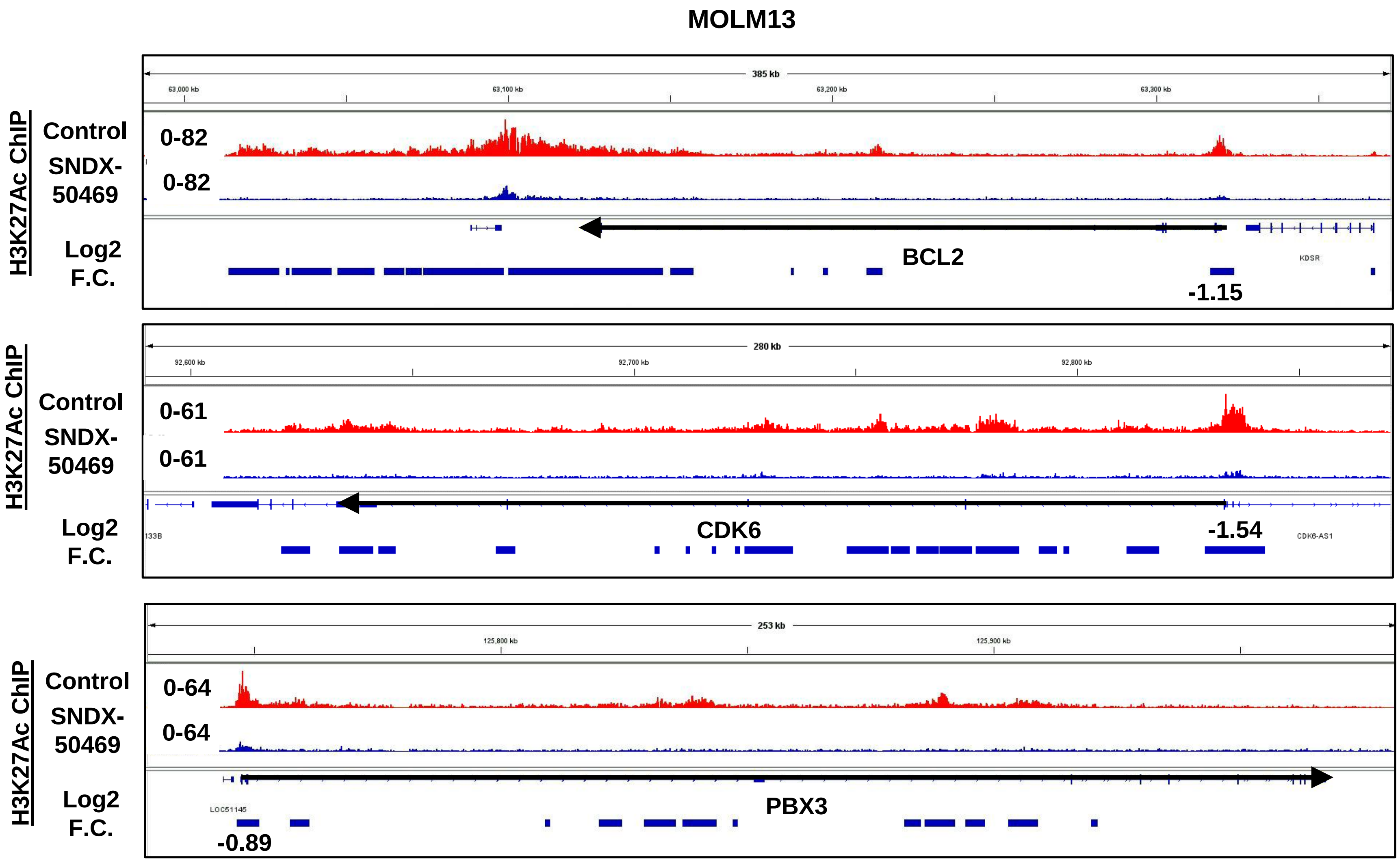
MOLM13
H3K27Ac ChIP
Control
SNDX-50469
Log2 F.C.
0-82
0-82
385 kb
63,000 kb
63,100 kb
63,200 kb
63,300 kb
BCL2
KDSR
-1.15
280 kb
92,600 kb
92,700 kb
92,800 kb
0-61
0-61
CDK6
CDK6-AS1
-1.54
253 kb
125,800 kb
125,900 kb
0-64
0-64
LOC51145
PBX3
-0.89

Figure S3

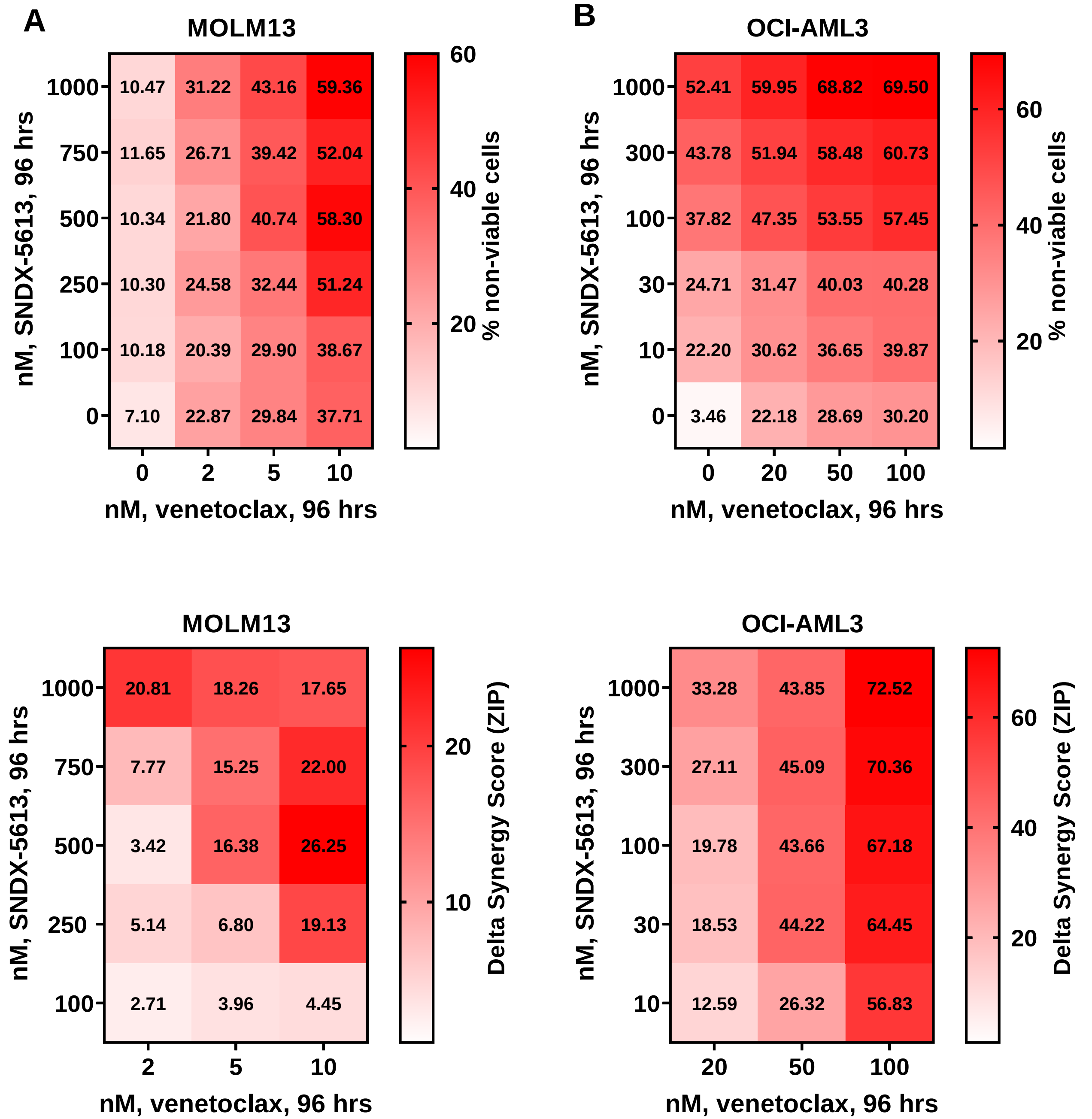
A
MOLM13
1000 10.47 31.22 43.16 59.36
750 11.65 26.71 39.42 52.04
500 10.34 21.80 40.74 58.30
250 10.30 24.58 32.44 51.24
100 10.18 20.39 29.90 38.67
0 7.10 22.87 29.84 37.71
0 2 5 10
nM, venetoclax, 96 hrs
nM, SNDX-5613, 96 hrs
% non-viable cells
60
40
20
B
OCI-AML3
1000 52.41 59.95 68.82 69.50
300 43.78 51.94 58.48 60.73
100 37.82 47.35 53.55 57.45
30 24.71 31.47 40.03 40.28
10 22.20 30.62 36.65 39.87
0 3.46 22.18 28.69 30.20
0 20 50 100
nM, venetoclax, 96 hrs
nM, SNDX-5613, 96 hrs
% non-viable cells
60
40
20
MOLM13
1000 20.81 18.26 17.65
750 7.77 15.25 22.00
500 3.42 16.38 26.25
250 5.14 6.80 19.13
100 2.71 3.96 4.45
2 5 10
nM, venetoclax, 96 hrs
nM, SNDX-5613, 96 hrs
Delta Synergy Score (ZIP)
20
10
OCI-AML3
1000 33.28 43.85 72.52
300 27.11 45.09 70.36
100 19.78 43.66 67.18
30 18.53 44.22 64.45
10 12.59 26.32 56.83
20 50 100
nM, venetoclax, 96 hrs
nM, SNDX-5613, 96 hrs
Delta Synergy Score (ZIP)
60
40
20

Figure S4

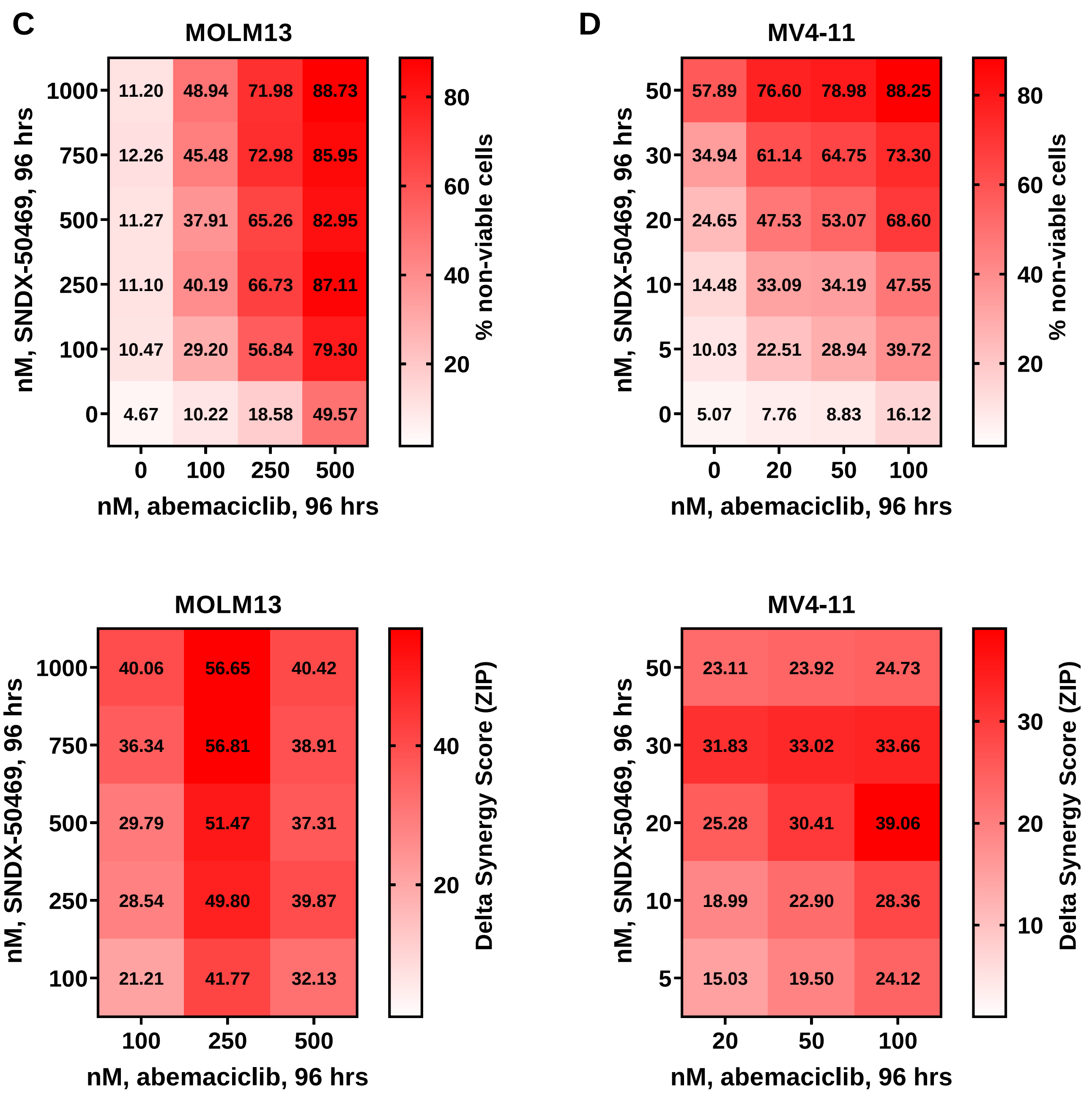
C
MOLM13
nM, SNDX-50469, 96 hrs
1000 11.20 48.94 71.98 88.73
750 12.26 45.48 72.98 85.95
500 11.27 37.91 65.26 82.95
250 11.10 40.19 66.73 87.11
100 10.47 29.20 56.84 79.30
0 4.67 10.22 18.58 49.57
0 100 250 500
nM, abemaciclib, 96 hrs
80 60 40 20
% non-viable cells
D
MV4-11
nM, SNDX-50469, 96 hrs
50 57.89 76.60 78.98 88.25
30 34.94 61.14 64.75 73.30
20 24.65 47.53 53.07 68.60
10 14.48 33.09 34.19 47.55
5 10.03 22.51 28.94 39.72
0 5.07 7.76 8.83 16.12
0 20 50 100
nM, abemaciclib, 96 hrs
80 60 40 20
% non-viable cells
MOLM13
nM, SNDX-50469, 96 hrs
1000 40.06 56.65 40.42
750 36.34 56.81 38.91
500 29.79 51.47 37.31
250 28.54 49.80 39.87
100 21.21 41.77 32.13
100 250 500
nM, abemaciclib, 96 hrs
40 20
Delta Synergy Score (ZIP)
MV4-11
nM, SNDX-50469, 96 hrs
50 23.11 23.92 24.73
30 31.83 33.02 33.66
20 25.28 30.41 39.06
10 18.99 22.90 28.36
5 15.03 19.50 24.12
20 50 100
nM, abemaciclib, 96 hrs
30 20 10
Delta Synergy Score (ZIP)

Figure S4

E

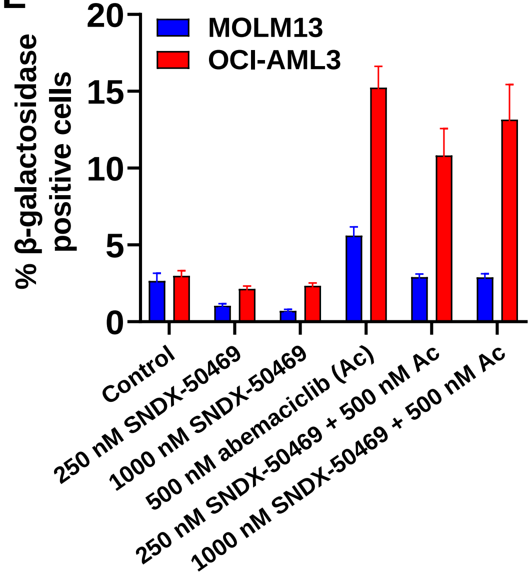

F

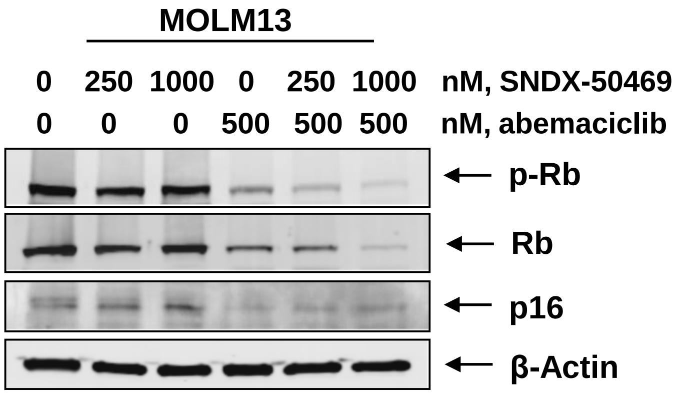

G

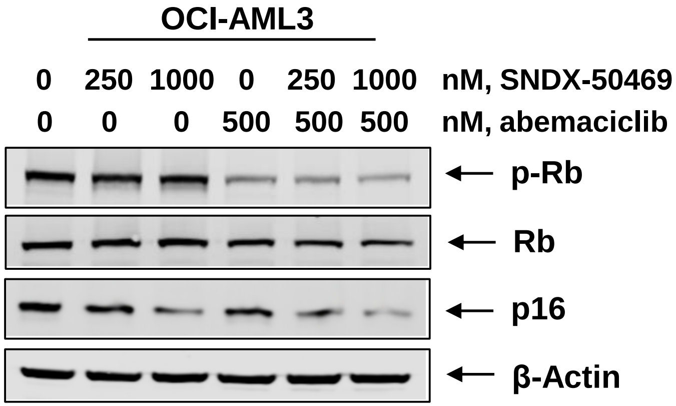

Figure S4

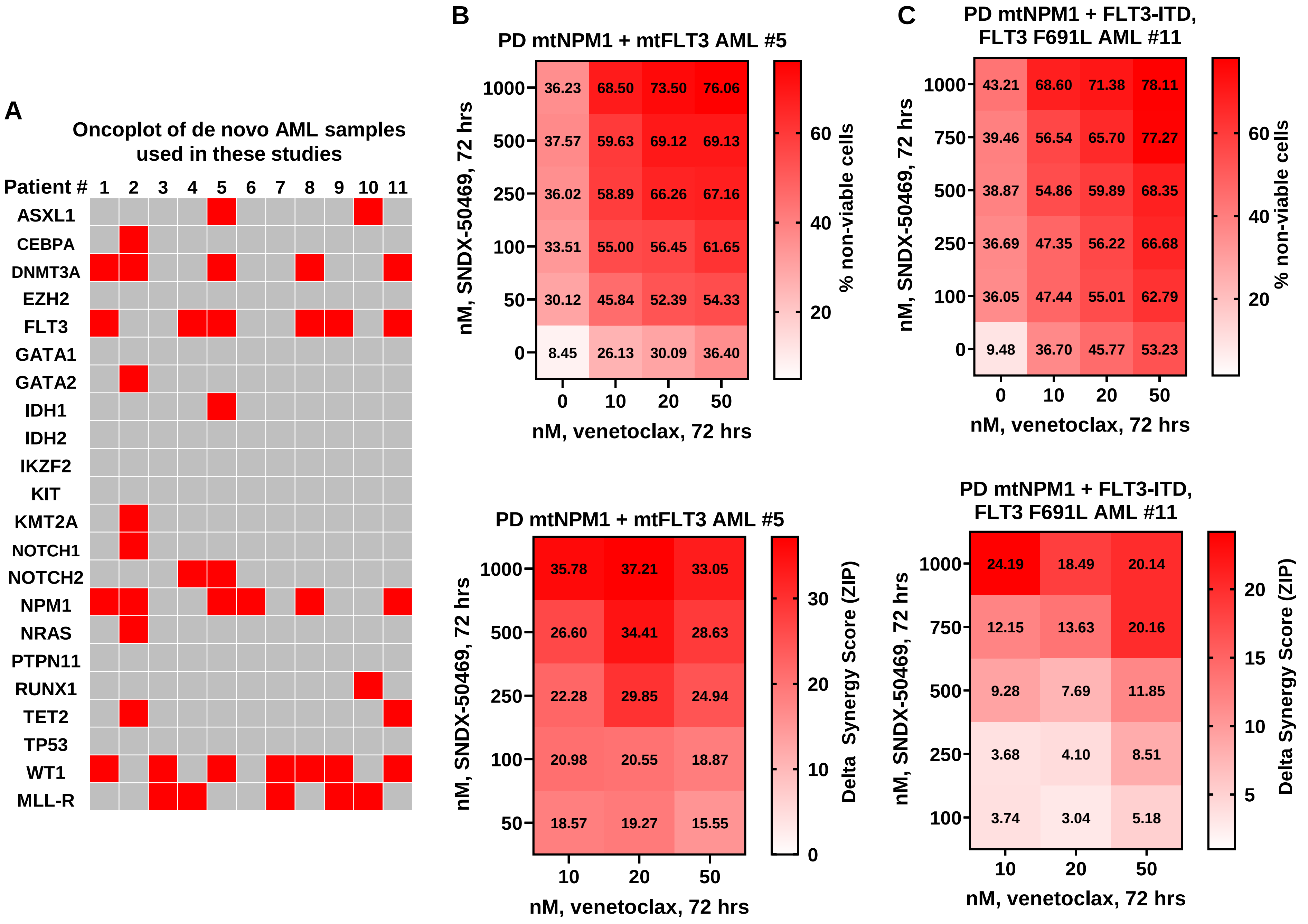
A
Oncoplot of de novo AML samples used in these studies
Patient # 1 2 3 4 5 6 7 8 9 10 11
ASXL1
CEBPA
DNMT3A
EZH2
FLT3
GATA1
GATA2
IDH1
IDH2
IKZF2
KIT
KMT2A
NOTCH1
NOTCH2
NPM1
NRAS
PTPN11
RUNX1
TET2
TP53
WT1
MLL-R
B
PD mtNPM1 + mtFLT3 AML #5
nM, SNDX-50469, 72 hrs
1000 500 250 100 50 0
36.23 68.50 73.50 76.06
37.57 59.63 69.12 69.13
36.02 58.89 66.26 67.16
33.51 55.00 56.45 61.65
30.12 45.84 52.39 54.33
8.45 26.13 30.09 36.40
0 10 20 50
nM, venetoclax, 72 hrs
% non-viable cells
20 40 60
C
PD mtNPM1 + FLT3-ITD, FLT3 F691L AML #11
nM, SNDX-50469, 72 hrs
1000 750 500 250 100 0
43.21 68.60 71.38 78.11
39.46 56.54 65.70 77.27
38.87 54.86 59.89 68.35
36.69 47.35 56.22 66.68
36.05 47.44 55.01 62.79
9.48 36.70 45.77 53.23
0 10 20 50
nM, venetoclax, 72 hrs
% non-viable cells
20 40 60
PD mtNPM1 + mtFLT3 AML #5
nM, SNDX-50469, 72 hrs
1000 500 250 100 50
35.78 37.21 33.05
26.60 34.41 28.63
22.28 29.85 24.94
20.98 20.55 18.87
18.57 19.27 15.55
10 20 50
nM, venetoclax, 72 hrs
Delta Synergy Score (ZIP)
0 10 20 30
PD mtNPM1 + FLT3-ITD, FLT3 F691L AML #11
nM, SNDX-50469, 72 hrs
1000 750 500 250 100
24.19 18.49 20.14
12.15 13.63 20.16
9.28 7.69 11.85
3.68 4.10 8.51
3.74 3.04 5.18
10 20 50
nM, venetoclax, 72 hrs
Delta Synergy Score (ZIP)
5 10 15 20

Figure S5

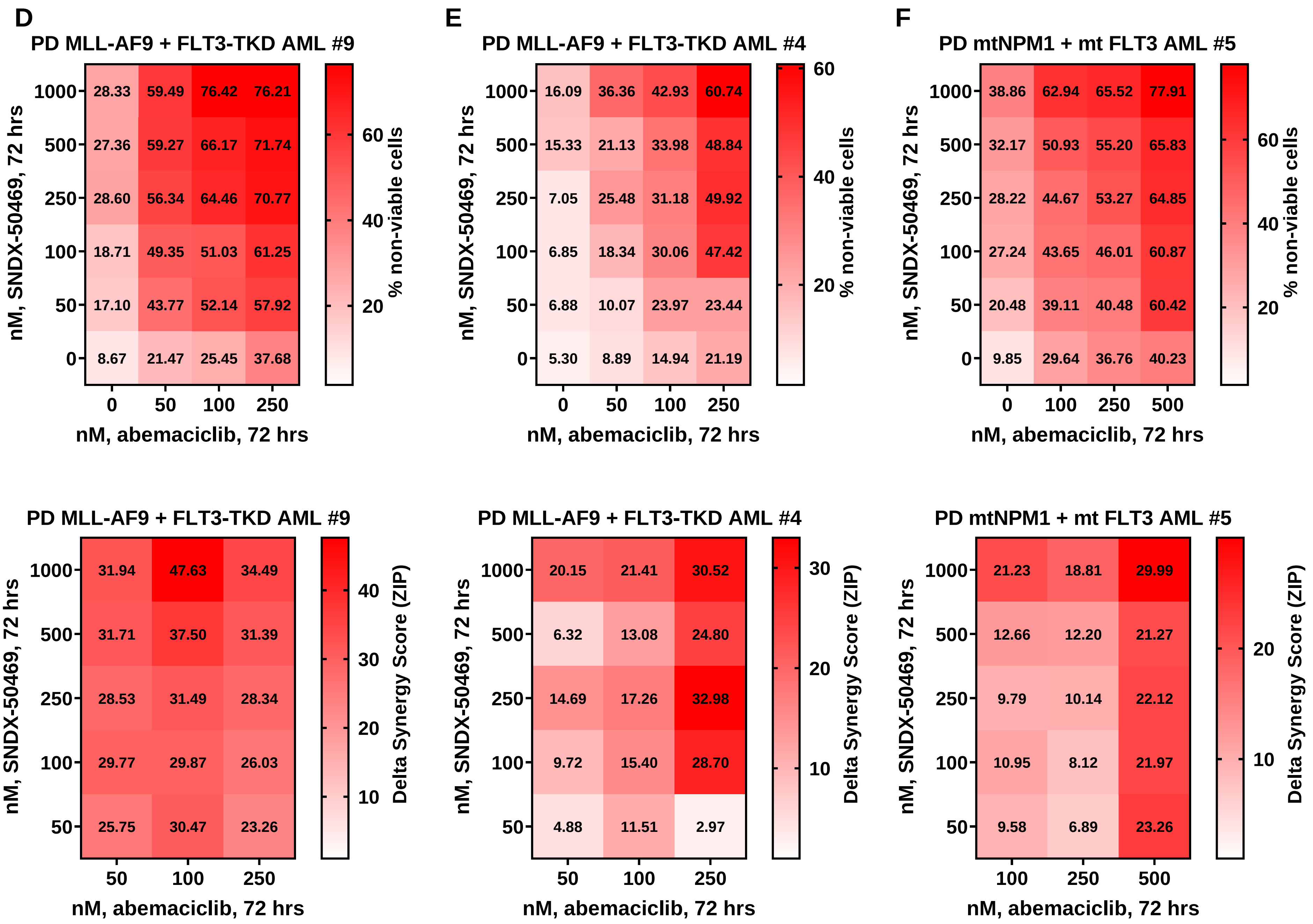
D
PD MLL-AF9 + FLT3-TKD AML #9
nM, SNDX-50469, 72 hrs
1000 28.33 59.49 76.42 76.21
500 27.36 59.27 66.17 71.74
250 28.60 56.34 64.46 70.77
100 18.71 49.35 51.03 61.25
50 17.10 43.77 52.14 57.92
0 8.67 21.47 25.45 37.68
0 50 100 250
nM, abemaciclib, 72 hrs
% non-viable cells
20 40 60
E
PD MLL-AF9 + FLT3-TKD AML #4
nM, SNDX-50469, 72 hrs
1000 16.09 36.36 42.93 60.74
500 15.33 21.13 33.98 48.84
250 7.05 25.48 31.18 49.92
100 6.85 18.34 30.06 47.42
50 6.88 10.07 23.97 23.44
0 5.30 8.89 14.94 21.19
0 50 100 250
nM, abemaciclib, 72 hrs
% non-viable cells
20 40 60
F
PD mtNPM1 + mt FLT3 AML #5
nM, SNDX-50469, 72 hrs
1000 38.86 62.94 65.52 77.91
500 32.17 50.93 55.20 65.83
250 28.22 44.67 53.27 64.85
100 27.24 43.65 46.01 60.87
50 20.48 39.11 40.48 60.42
0 9.85 29.64 36.76 40.23
0 100 250 500
nM, abemaciclib, 72 hrs
% non-viable cells
20 40 60
PD MLL-AF9 + FLT3-TKD AML #9
nM, SNDX-50469, 72 hrs
1000 31.94 47.63 34.49
500 31.71 37.50 31.39
250 28.53 31.49 28.34
100 29.77 29.87 26.03
50 25.75 30.47 23.26
50 100 250
nM, abemaciclib, 72 hrs
Delta Synergy Score (ZIP)
10 20 30 40
PD MLL-AF9 + FLT3-TKD AML #4
nM, SNDX-50469, 72 hrs
1000 20.15 21.41 30.52
500 6.32 13.08 24.80
250 14.69 17.26 32.98
100 9.72 15.40 28.70
50 4.88 11.51 2.97
50 100 250
nM, abemaciclib, 72 hrs
Delta Synergy Score (ZIP)
10 20 30
PD mtNPM1 + mt FLT3 AML #5
nM, SNDX-50469, 72 hrs
1000 21.23 18.81 29.99
500 12.66 12.20 21.27
250 9.79 10.14 22.12
100 10.95 8.12 21.97
50 9.58 6.89 23.26
100 250 500
nM, abemaciclib, 72 hrs
Delta Synergy Score (ZIP)
10 20

Figure S5

G

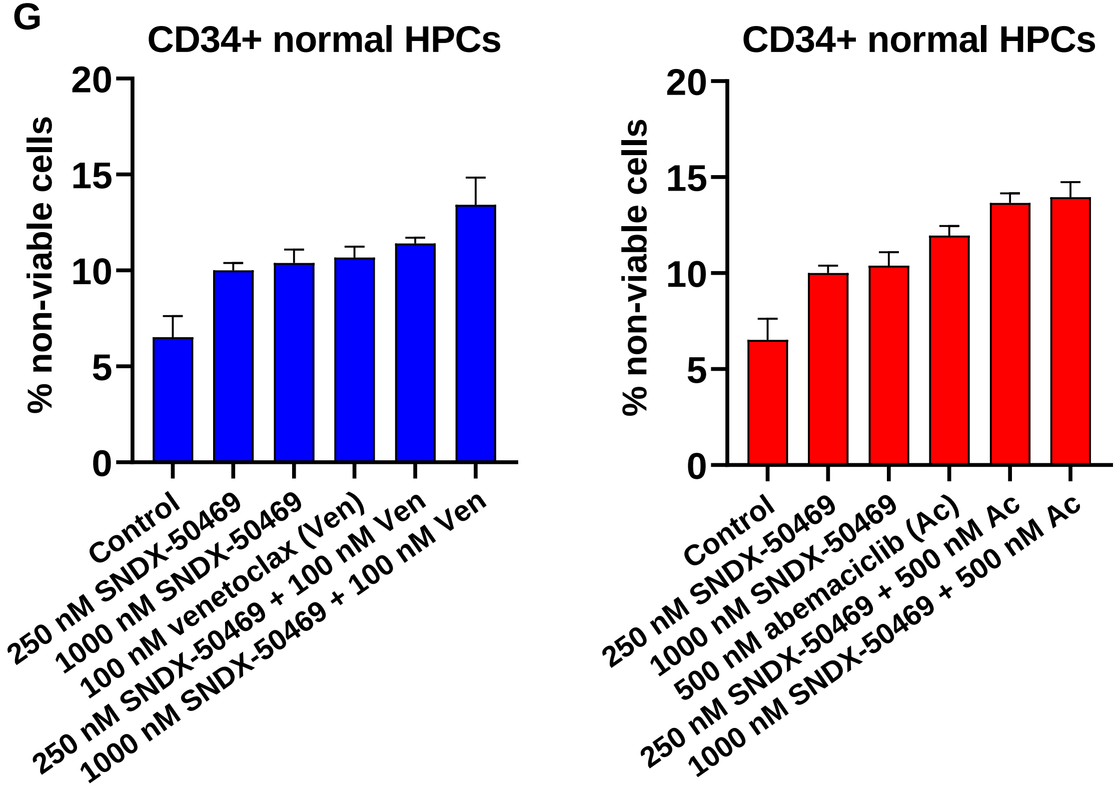
CD34+ normal HPCs
% non-viable cells
20
15
10
5
0
Control
250 nM SNDX-50469
1000 nM SNDX-50469
100 nM venetoclax (Ven)
250 nM SNDX-50469 + 100 nM Ven
1000 nM SNDX-50469 + 100 nM Ven
CD34+ normal HPCs
% non-viable cells
20
15
10
5
0
Control
250 nM SNDX-50469
1000 nM SNDX-50469
500 nM abemaciclib (Ac)
250 nM SNDX-50469 + 500 nM Ac
1000 nM SNDX-50469 + 500 nM Ac

Figure S5

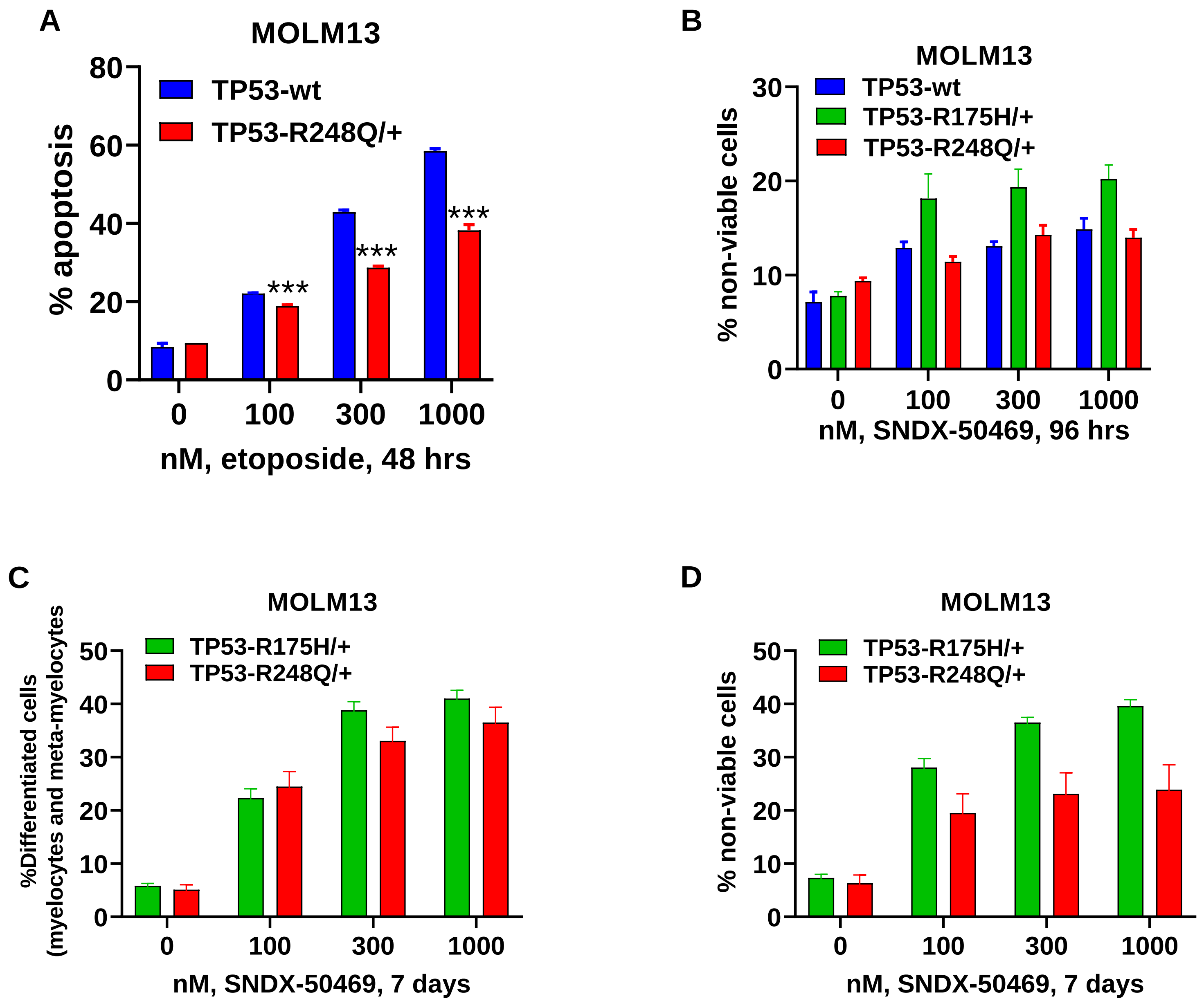
A
MOLM13
TP53-wt
TP53-R248Q/+
% apoptosis
80
60
40
20
0
***
***
***
0
100
300
1000
nM, etoposide, 48 hrs
B
MOLM13
TP53-wt
TP53-R175H/+
TP53-R248Q/+
% non-viable cells
30
20
10
0
0
100
300
1000
nM, SNDX-50469, 96 hrs
C
MOLM13
TP53-R175H/+
TP53-R248Q/+
%Differentiated cells
(myelocytes and meta-myelocytes
50
40
30
20
10
0
0
100
300
1000
nM, SNDX-50469, 7 days
D
MOLM13
TP53-R175H/+
TP53-R248Q/+
% non-viable cells
50
40
30
20
10
0
0
100
300
1000
nM, SNDX-50469, 7 days

Figure S6

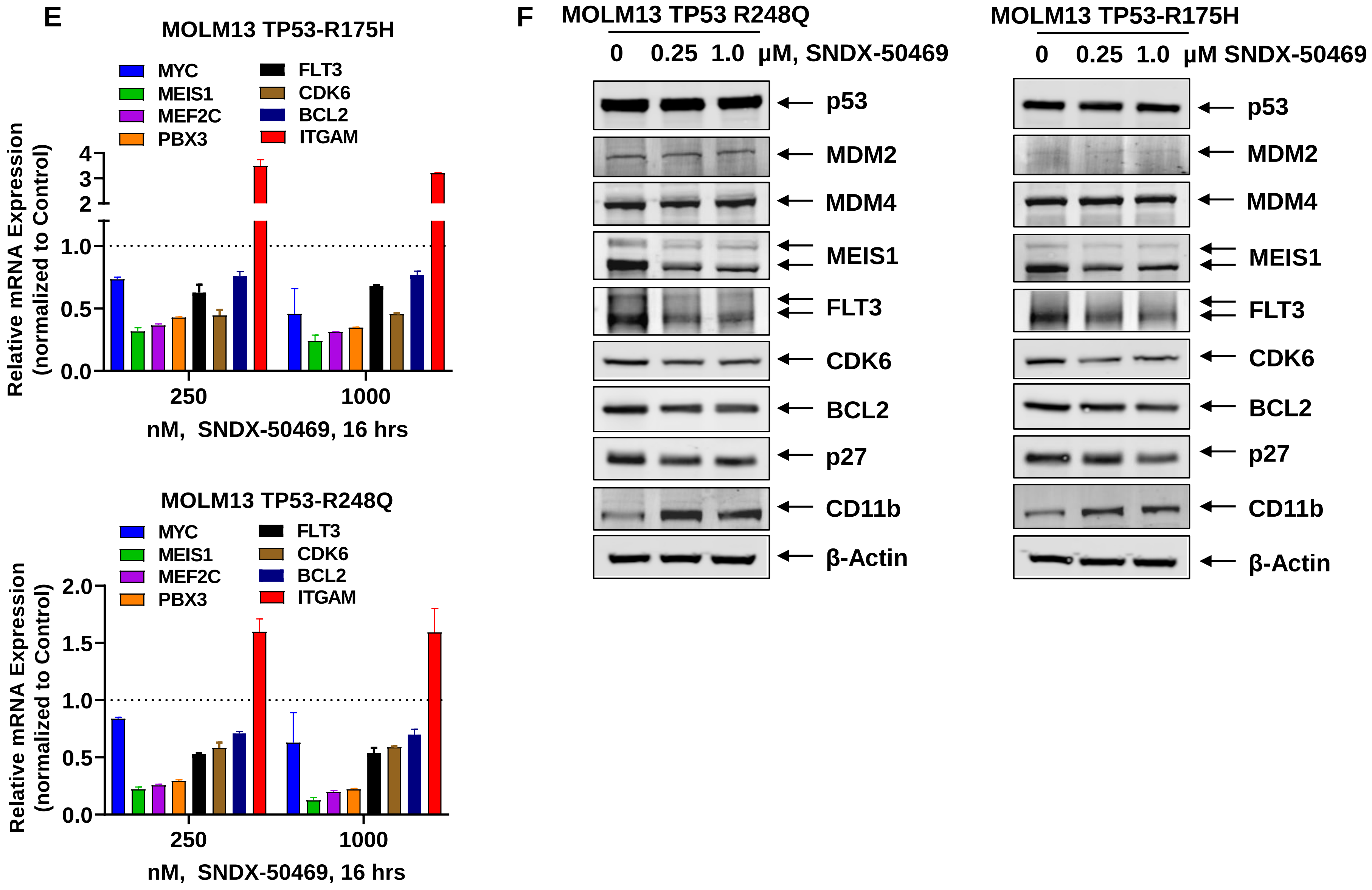
E
MOLM13 TP53-R175H
MYC
MEIS1
MEF2C
PBX3
FLT3
CDK6
BCL2
ITGAM
Relative mRNA Expression (normalized to Control)
4
3
2
1.0
0.5
0.0
250
1000
nM, SNDX-50469, 16 hrs
MOLM13 TP53-R248Q
MYC
MEIS1
MEF2C
PBX3
FLT3
CDK6
BCL2
ITGAM
Relative mRNA Expression (normalized to Control)
2.0
1.5
1.0
0.5
0.0
250
1000
nM, SNDX-50469, 16 hrs
F
MOLM13 TP53 R248Q
0 0.25 1.0 μM, SNDX-50469
MOLM13 TP53-R175H
0 0.25 1.0 μM SNDX-50469
p53
MDM2
MDM4
MEIS1
FLT3
CDK6
BCL2
p27
CD11b
β-Actin

Figure S6

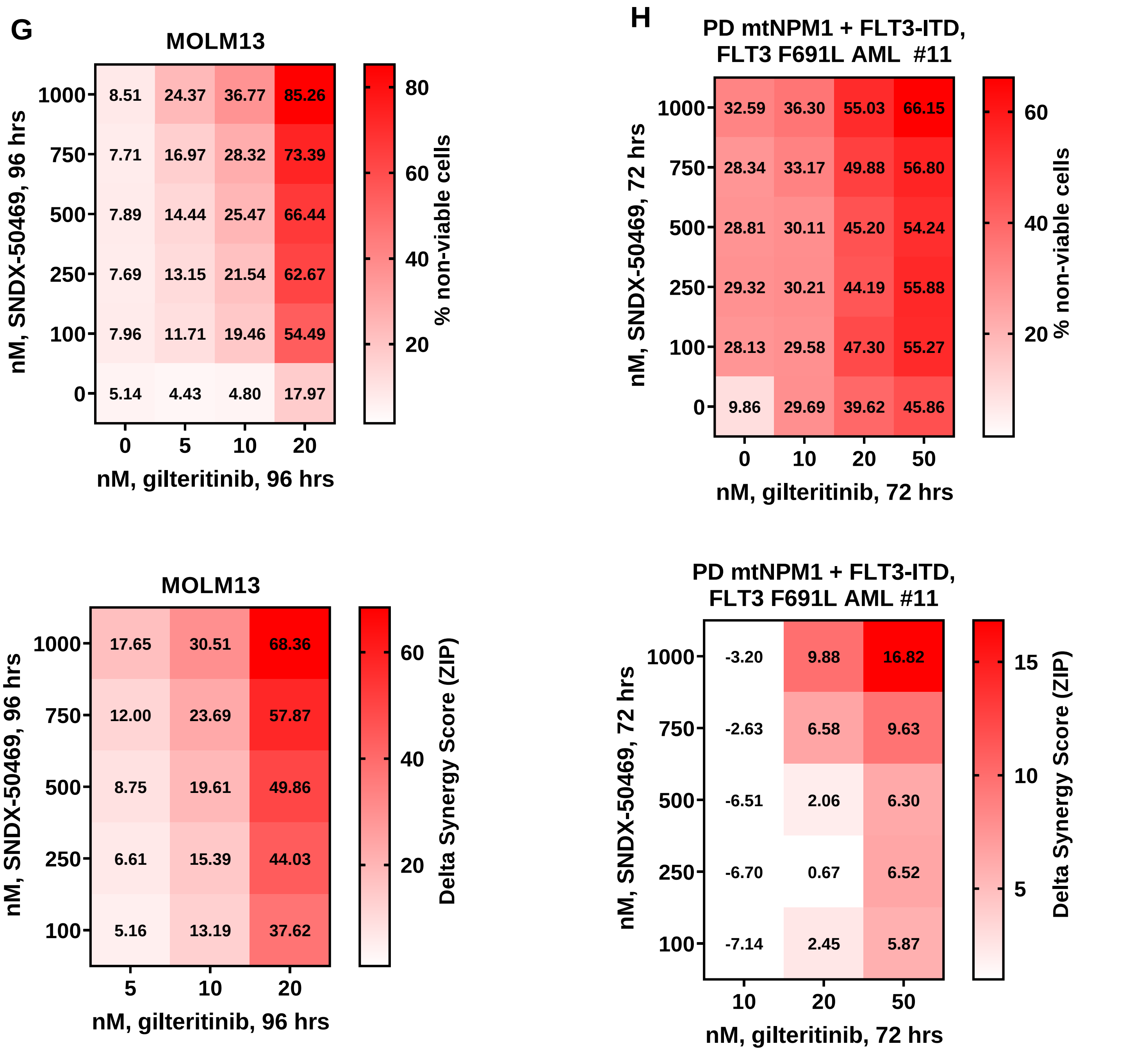
G
MOLM13
nM, SNDX-50469, 96 hrs
1000
750
500
250
100
0
8.51 24.37 36.77 85.26
7.71 16.97 28.32 73.39
7.89 14.44 25.47 66.44
7.69 13.15 21.54 62.67
7.96 11.71 19.46 54.49
5.14 4.43 4.80 17.97
0 5 10 20
nM, gilteritinib, 96 hrs
80
60
40
20
% non-viable cells
H
PD mtNPM1 + FLT3-ITD,
FLT3 F691L AML #11
nM, SNDX-50469, 72 hrs
1000
750
500
250
100
0
32.59 36.30 55.03 66.15
28.34 33.17 49.88 56.80
28.81 30.11 45.20 54.24
29.32 30.21 44.19 55.88
28.13 29.58 47.30 55.27
9.86 29.69 39.62 45.86
0 10 20 50
nM, gilteritinib, 72 hrs
60
40
20
% non-viable cells
MOLM13
nM, SNDX-50469, 96 hrs
1000
750
500
250
100
17.65 30.51 68.36
12.00 23.69 57.87
8.75 19.61 49.86
6.61 15.39 44.03
5.16 13.19 37.62
5 10 20
nM, gilteritinib, 96 hrs
60
40
20
Delta Synergy Score (ZIP)
PD mtNPM1 + FLT3-ITD,
FLT3 F691L AML #11
nM, SNDX-50469, 72 hrs
1000
750
500
250
100
-3.20 9.88 16.82
-2.63 6.58 9.63
-6.51 2.06 6.30
-6.70 0.67 6.52
-7.14 2.45 5.87
10 20 50
nM, gilteritinib, 72 hrs
15
10
5
Delta Synergy Score (ZIP)

Figure S6

Co-mutations in AML#4 PDX

Gene	Protein Change	Variant Allele Freq (VAF)
FLT3	N676T	0.917355
KMT2C	C988F	0.363392
KMT2C	Y987H	0.18362
KMT2C	D348N	0.078125
KMT2C	V919L	0.073446
KMT2C	S990G	0.07
KMT2C	G315S	0.04148
KMT2C	R380L	0.041427
KMT2C	R284Q	0.035762
KMT2C	C391*	0.033722
KMT2C	P309S	0.031927
KMT2D	P2107T	0.521739
NOTCH2	G225R	0.395604
NOTCH2	C19W	0.448276
ARID1A	L1724R	0.448276
IRF8	*427Yext*24	0.540881
ABCA7	P1503L	0.539519
VPS13A	S734N	0.620079

Table S1

Table S2. In 190 patients with MLL1-r AML managed at M.D. Anderson Cancer Center (2012-2021), concurrent TP53 mutations were identified in 17 patients (~9%).

Patient Number	Cytogenetic Alteration (MLL rearrangement)	Nucleotide alteration in TP53	Protein alteration in TP53
1	t(9;11)(p22;q23)	c.659A>G	p.Y220C
2	t(9;11)(p22;q23)	c.542G>C	p.R181P
3	t(5;11)(p14;q23)	c.399_406del	p.M133fs*13
4	t(4;11)(q21;q23)	c.845_860delins31	p.R282_E287delinsPSVGSLPE EEE
5	t(4;11)(q21;q23)	c.845_846insAATATG	p.R282_R283ins
6	t(4;11)(q21;q23)	c.524G>A	p.R175H
7	t(4;11)(q21;q23)	c.817C>A	p.R273S
8	t(4;11)(q21;q23)	c.845G>C	p.R282P
9	t(4;11)(q21;q23)	c.488A>C	p.Y163S
10	t(4;11)(q21;q23)	c.818G>A	p.R273H
11	t(11;19)(q23;q13.3)	c.536A>G	p.H179R
12	t(11;18)(q23;q21.3)	c.139C>T	p.P47S
13	t(11;16)(q23;p13.3)	c.743G>A	p.R248Q
14	t(11;14)(q23;q32)	c.425dupC	p.V143fs
15	der(6)t(6;11)(q27;q23)	c.811G>T	p.E271*
16	der(4)t(4;11)(q21;q23)	c.845G>C	p.R282P
17	der(12)t(11;12)(q23;q22)	c.414dupC	p.K139fs

Table S3

Co-mutations in AML# 5 PDX

Gene	Protein Change	Variant Allele Freq (VAF)
DNMT3A	R882H	0.487889
IDH1	R132H	0.484305
NPM1	W288Cfs*12	0.423358
FLT3	V194M	0.450135
WT1	H445Q	0.252137
KMT2C	R284Q	0.059818
KMT2C	P309S	0.138462
KMT2C	D348N	0.084453
KMT2C	C988F	0.217899
KMT2C	Y987H	0.270115
NOTCH2	C19W	0.416268
ZFHX3	G3517D	0.5625
PCLO	S496P	0.079848
CCND3	L132F	0.463158
CD36	G217R	0.495017
ATM	F858L	0.489914
CIITA	S299F	0.446602
ZFHX3	R3381_Q3387del	0.33119
NF1	V1453A	0.478814
STAT5A	P194L	0.477733
RNF213	S2334N	0.995122
SMARCA4	V720M	0.182266
ASXL1	G704R	0.498818
GNAS	R1023G	0.152589
ABCA7	A799E	0.4875
