# Supplementary material for: Effective Menin inhibitor-based combinations against AML with MLL rearrangement or NPM1 mutation (NPM1c)
Source: Blood Cancer J. 2022 Jan 11;12(1):5. doi: 10.1038/s41408-021-00603-3 (PMC8752621; doi:10.1038/s41408-021-00603-3)
Supplement: Supplementary file 2 — Supplemental Figure Legends [file 41408_2021_603_MOESM2_ESM.docx]

**Supplemental Figure Legends**

**Figure S1. Co-treatment with MEK inhibitor destabilizes MCL1 expression in Menin inhibitor-treated AML cells**. **A**. Quantification of protein expression alterations in MOLM13 cells with knockout of Menin by CRISPR-Cas9-mediated gene editing compared to sgNeg-transfected MOLM13 cells. Columns; mean of two independent experiments + S.D. * =p<0.05, ** =p< 0.01, *** =p< 0.005 compared to MOLM13 sgNeg-transfected cells (determined by a two-tailed, unpaired t-test). **B**. Quantification of protein expression alterations in MOLM13 Menin-FKBP12^(F36V)^ cells treated with the indicated concentrations of dTAG-13 for 72 hours compared to untreated cells. Columns; mean of two independent experiments + S.D. * =p<0.05, ** = p< 0.01, *** = p< 0.005 compared to untreated MOLM13 cells (determined by a two-tailed, unpaired t-test). **C**. Chemical structure for SNDX-50469 and SNDX-5613. **D**. Quantification of protein expression alterations in MOLM13 and OCI-AML3 cells treated with SNDX-50469 for 48 hours. Columns, mean of two independent experiments + S.D. * =p<0.05, ** = p< 0.01, *** = p< 0.005 compared to untreated MOLM13 or OCI-AMl3 cells (determined by a two-tailed, unpaired t-test). **E**. OCI-AML3 cells were treated with the indicated concentrations of SNDX-50469 for 48 hours. Total cell lysates were prepared and immunoblot analyses were conducted. The expression levels of β-Actin in the lysates served as the loading control. **F**. OCI-AML3 cells were treated with the indicated concentration of SNDX-50469 for 48 hours, without or with 250 nM of MEK inhibitor cobimetinib (added in the last 24 hours of treatment). Total cell lysates were prepared and immunoblot analyses were conducted. **G**. OCI-AML3 cells were treated with the indicated concentrations of SNDX-50469, without or with 250 nM of MEK inhibitor cobimetinib for 48 hours. Then, the % of annexin-V positive, apoptotic cells were determined by flow cytometry. Columns, mean of 3 experiments + S.E.M. *** = p< 0.005; (determined by two-tailed, unpaired t-test in GraphPad V8).

**Figure S2. Treatment with Menin inhibitor SNDX-50469 induces features of morphologic differentiation in AML cells**. **A**. MV4-11 cells were treated with the indicated concentrations of SNDX-50469 for 96 hours. Morphologic differentiation (% myelocytes, meta-myelocytes or bands) was determined by light microscopy. Mean of three experiments + S.D. **B**. Representative images of control and SNDX-50469-induced morphologic differentiation in MOLM13, OCI-AML3 and MV4-11 cells. **C**. MOLM13 cells were treated with the indicated concentrations of SNDX-50469 for 7 days. At the end of treatment, the % of TO-PRO-3 iodide-positive, non-viable cells were determined by flow cytometry. Columns, mean of 3 experiments + S.E.M.

**Figure S3. Menin inhibitor treatment depletes H3K27 acetyl mark on the enhancers and promoters of BCL2, CDK6 and PBX3 in cultured AML blasts**. IGV plots showing the signal tag density of H3K27Ac ChIP-Seq on the BCL2, CDK6 and PBX3 gene in MOLM13 cells treated with SNDX-50469 for 16 hours. Black arrows mark the direction of the coding sequence of each gene. Blue bars beneath the gene indicate significant (p < 0.05) log2 fold-changes in signal tag density of H3K27Ac in SNDX-50469-treated cells compared to control cells.

**Figure S4. Co-treatment with Menin inhibitor and venetoclax or CDK4/6 inhibitor abemaciclib exerts synergistic in vitro lethality in cultured AML cells**. **A-B**. MOLM13 and OCI-AML3 cells were treated with the indicated concentrations of SNDX-5613 and/or venetoclax for 96 hours. At the end of treatment, the % non-viable cells were determined by staining with TO-PRO-3 iodide and flow cytometry analysis. Delta synergy scores were determined by the ZIP method. Synergy scores >1.0 indicate a synergistic interaction of the two agents in the combination. **C-D**. MOLM13 and MV4-11 cells were treated with the indicated concentrations of SNDX-50469 and/or abemaciclib for 96 hours. At the end of treatment, the % non-viable cells were determined by staining with TO-PRO-3 iodide and flow cytometry analysis. Delta synergy scores were determined by the ZIP method. Synergy scores >1.0 indicate a synergistic interaction of the two agents in the combination. **E**. MOLM13 and OCI-AML3 cells were treated with the indicated concentrations of SNDX-50469 and/or abemaciclib for 48 hours. At the end of treatment, induction of senescence (as detected by activation of β-galactosidase production) was determined utilizing the CellEvent^TM^ Senescence Green Assay kit and flow cytometry. Columns, mean of 2 experiments performed in duplicate + S.E.M. **F-G**. MOLM13 and OCI-AML3 cells were treated with the indicated concentrations of SNDX-50469 and/or abemaciclib for 48 hours. Total cell lysates were prepared and immunoblot analyses were conducted as indicated.

**Figure S5. Co-treatment with Menin inhibitor and venetoclax or CDK4/6 inhibitor abemaciclib induces synergistic in vitro lethality in patient-derived AML cells expressing mtNPM1 with or without FLT3 alterations while sparing CD34+ normal hematopoietic progenitor cells (HPCs). A.** Oncoplot of the mutations identified in patient-derived AML cells utilized in these studies. Mutations identified by next generation sequencing of a custom panel of 81 genes. MLL rearrangement was confirmed by cytogenetic analysis. **B-C**. Patient-derived AML cells with MLL rearrangement or mtNPM1 with or without FLT3 alterations were treated with the indicated concentrations of SNDX-50469 and/or venetoclax for 48-72 hours. The % non-viable cells were determined by staining with TO-PRO-3 iodide and flow cytometry analysis. Delta synergy scores were determined by the ZIP method. Synergy scores >1.0 indicate a synergistic interaction of the two agents in the combination. **D-F**. Primary AML cells with MLL1-r and mtFLT3 or mtNPM1 and mtFLT3 were treated with the indicated concentrations of SNDX-50469 and/or abemaciclib for 96 hours. At the end of treatment, the % non-viable cells were determined by staining with TO-PRO-3 iodide and flow cytometry analysis. Delta synergy scores were determined by the ZIP method. Synergy scores >1.0 indicate a synergistic interaction of the two agents in the combination. **G**. CD34+ normal HPCs (n=3) were treated with the indicated concentrations of SNDX-50469 and/or venetoclax (Ven) or abemaciclib (Ac) for 72 hours. Following this, the % non-viable cells were determined by staining with TO-PRO-3 iodide and flow cytometry analysis.

**Figure S6.** **Menin inhibitor treatment depletes mRNA and protein expressions of MLL targets in MOLM13 cells with isogenic TP53 mutations and co-treatment with Menin inhibitor and FLT3 inhibitor gilteritinib induces synergistic in vitro lethality in MOLM13 cells and PD AML cells expressing mtNPM1 and FLT3 alterations**. **A**. MOLM13 and MOLM13 TP53-R248Q/+ cells were treated with the indicated concentrations of etoposide for 48 hours. At the end of treatment, cells were stained with annexin V-FITC and TO-PRO-3 iodide and the % of annexin V-positive, apoptotic cells were determined by flow cytometry. Columns, mean of 3 experiments + S.E.M. *** = p< 0.005; (determined by two-tailed, unpaired t-test in GraphPad V8). **B**. MOLM13, MOLM13-TP53-R175H/+ and MOLM13 TP53-R248Q/+ cells were treated with the indicated concentrations of SNDX-50469 for 96 hours. Then, the % non-viable cells were determined by staining with TO-PRO-3 iodide and flow cytometry analysis. Columns, mean of 3 experiments + S.E.M. **C**. MOLM13-TP53-R175H/+ and MOLM13 TP53-R248Q/+ cells were treated with the indicated concentrations of SNDX-50469 for 7 days. Then, morphologic differentiation (% myelocytes, meta-myelocytes or bands) was determined by light microscopy. Columns, mean of 3 experiments + S.E.M. **D.** MOLM13-TP53-R175H/+ and MOLM13 TP53-R248Q/+ cells were treated with the indicated concentrations of SNDX-50469 for 7 days. Then, the % non-viable cells were determined by staining with TO-PRO-3 iodide and flow cytometry analysis. Columns, mean of 3 experiments + S.E.M. **E**. MOLM13 TP53-R175H and MOLM13 TP53-R248Q cells were treated with the indicated concentrations of SNDX-50469 for 16 hours. Total RNA was isolated and reverse transcribed. The resulting cDNAs were used for qPCR as shown. The expression of GAPDH served as the normalization control. **F**. MOLM13 TP53-R175H and MOLM13 TP53-R248Q cells were treated with the indicated concentrations of SNDX-50469 for 48 hours. Total cell lysates were prepared and immunoblot analyses were conducted. The expression levels of β-Actin in the lysates served as the loading control. **G**. MOLM13 cells were treated with the indicated concentrations of SNDX-50469 and/or gilteritinib for 96 hours. At the end of treatment, the % non-viable cells were determined by staining with TO-PRO-3 iodide and flow cytometry analysis. Delta synergy scores were determined by the ZIP method. Synergy scores >1.0 indicate a synergistic interaction of the two agents in the combination. **H**. PD, mtNPM1 + FLT3-ITD + FLT3 F691L AML cells were treated with the indicated concentrations of SNDX-50469 and/or gilteritinib for 72 hours. The % non-viable cells were determined by staining with TO-PRO-3 iodide and flow cytometry analysis. Delta synergy scores were determined by the ZIP method. Synergy scores >1.0 indicate a synergistic interaction of the two agents in the combination.

**Table S1. Co-mutations and their associated variant allele frequency (%VAF) identified in the MLL-AF9 + FLT3-TKD PDX (AML#4) utilized in this study.**

**Table S2. In 190 patients with MLL1-r AML managed at M.D. Anderson Cancer Center (2012-2021), concurrent TP53 mutations were identified in 17 patients (~9%).**

**Table S3. Co-mutations and their associated variant allele frequency (%VAF) in the mtNPM1 and mtFLT3 PDX (AML#5) utilized for *in vivo* analysis of combined treatment with SNDX-5613 and venetoclax.**
