# Supplementary material for: Effective Menin inhibitor-based combinations against AML with MLL rearrangement or NPM1 mutation (NPM1c)
Source: Blood Cancer J. 2022 Jan 11;12(1):5. doi: 10.1038/s41408-021-00603-3 (PMC8752621; doi:10.1038/s41408-021-00603-3)
Supplement: Supplementary file 3 — Supplemental Materials and Methods [file 41408_2021_603_MOESM3_ESM.docx]

**Supplemental Materials and Methods:**

**Contact for Reagent sharing. Kapil N Bhalla. Department of Leukemia, MD. Anderson Cancer Center, 1400 Holcombe Blvd, Unit428, Houston, TX, 77030. kbhalla@mdanderson.org**

**Reagents and antibodies.** SNDX-50469, venetoclax, gilteritinib, cobimetinib, etoposide, and abemaciclib were obtained from MedChem Express (Monmouth Junction, NJ). DTAG-13 (Cat. No. 6605) was obtained from Tocris/Bio-Techne (Minneapolis, MN). SNDX-5613 was obtained from Syndax Pharmaceuticals Inc. (Waltham, MA) under a material transfer agreement. All compounds were prepared as 10 mM stocks in 100% DMSO and frozen at -80°C in 5-10 µL aliquots to allow for single use, thus avoiding multiple freeze-thaw cycles that could result in compound decomposition and loss of activity. Anti-c-Myc [RRID:AB_1903938], anti-MCL1 [RRID:AB_2799149], anti-BIM [RRID:AB_1030947], anti-BAK [RRID:AB_10828597], anti-BAX [RRID:AB_10557411], anti-PUMA [RRID:AB_2797920], anti-Bcl-xL [RRID:AB_10695729], anti-Menin [RRID:AB_10858216], anti-β-Tubulin [#86298], anti-MEF2C [RRID:AB_10548759], anti-p-ERK1/2 [RRID:AB_331772], ERK1/2 [RRID:AB_390779], p-S6 [RRID:AB_916156], total S6 [RRID:AB_331355], p-Rb [RRID:AB_11178658], total Rb [RRID:AB_823629], and p16 [RRID:AB_2799960] antibodies were obtained from Cell Signaling Technologies (Beverly, MA). Anti-HA-tag [RRID:AB_444303], Anti-MEIS1 [RRID:AB_776272], anti-FLT3 [ab245116], anti-HOXA9 [ab140631], anti-PBX3 [RRID:AB_10858991], and anti-CD11b [RRID:AB_2650514] antibodies were obtained from Abcam (Cambridge, MA). Anti-BFL1 [# ABC490] antibody was obtained from Millipore/Sigma (Burlington, MA). Anti-CDK6 [RRID:AB_10610066], anti-BCL2 [RRID:AB_626733], anti-NOXA [RRID:AB_784877], anti-GAPDH [RRID:AB_627679] and anti-β-Actin [RRID:AB_626630] antibodies were obtained from Santa Cruz Biotechnologies (Santa Cruz, CA). Anti-p27 [RRID:AB_397636] antibody was obtained from BD Transduction Labs (Franklin Lakes, NJ).

**Cell lines and cell culture.** MOLM13 [DSMZ Cat# ACC-554, RRID: CVCL_2119] and OCI-AML3 [DSMZ Cat# ACC-582, RRID:CVCL_1844] cells were obtained from the DSMZ. MV4-11 [ATCC Cat# CRL-9591, RRID:CVCL_0064], cells were obtained from the ATCC (Manassas, VA). MOLM13 cells with isogenic TP53 mutations [R175H, R248Q and TP53-KO] were a gift from Dr. Benjamin L. Ebert (Dana Farber Cancer Center, Boston, MA). HEK-293T cells were obtained from the Characterized Cell Line Core Facility at M.D. Anderson Cancer Center, Houston TX. All experiments with cell lines were performed within 6 months after thawing or obtaining from ATCC or DSMZ. The cell lines were also authenticated in the Characterized Cell Line Core Facility at M.D. Anderson Cancer Center, Houston TX. MOLM13 and OCI-AML3 cells were cultured in RPMI-1640 media with 20% FBS and 1% penicillin/streptomycin. MV4-11 cells were cultured in ATCC-formulated IMDM media with 20% FBS and 1% penicillin/streptomycin. HEK293T cells were cultured in high-glucose-formulated DMEM media with 10% FBS, 1% penicillin/streptomycin and 1% glutamine. Logarithmically growing, mycoplasma-negative cells were utilized for all experiments. Following drug treatments, cells were washed free of the drug(s) prior to the performance of the studies described.

**Cell Line Authentication**. The cell lines utilized in these studies were authenticated in the Characterized Cell Line Core Facility at M.D. Anderson Cancer Center, Houston TX utilizing STR profiling.

**Primary AML blasts:** Patient-derived AML cells samples were obtained with informed consent as part of a clinical protocol approved by the Institutional Review Board of The University of Texas, M.D. Anderson Cancer Center. Mononuclear cells were purified by Ficoll Hypaque (Axis Shield, Oslo, Norway) density centrifugation following the manufacturer’s protocol. Mononuclear cells were washed once with sterile 1X PBS then suspended in complete RPMI media containing 20% FBS. Cells were counted to determine the number of cells isolated prior to immuno-magnetic selection. CD34+ AML blast progenitor cells were purified by immuno-magnetic beads conjugated with anti-CD34 antibody following the manufacturer’s protocol (StemCell Technologies, Vancouver, British Columbia) prior to utilization in the cell viability assays, RNA expression, and immunoblot analyses.

**Sequencing of primary de novo blast cells:** We performed targeted next-generation sequencing (NGS) of DNA samples from bone marrow or peripheral blood collected from patients at our center with de novo AML (1). Diagnostic bone marrow samples were obtained for mutational analysis. Total genomic DNA was extracted from unenriched peripheral blood (PB) or bone marrow (BM) samples using ReliaPrep genomic DNA isolation kit (Promega Corp, Madison, WI, USA). Briefly, a total of 250 ng DNA was utilized to prepare sequencing libraries using Agilent HaloPlex custom Kit (Agilent Technologies, Santa Clara, CA, USA). The entire coding sequences of 81 genes including ABL1, ASXL1, BRAF, CALR, DNMT3A, EGFR, EZH2, FLT3, GATA1, GATA2, HRAS, IDH1, IDH2, KIT, KRAS, MDM2, IKZF2, JAK1, JAK2, MLL, MPL, MYD88, NOTCH1, NF1, NPM1, NRAS, PTPN11, RUNX1, TET2, TP53, and WT1 were interrogated on a custom-designed next-generation sequencing approach using the Illumina MiSeq platform [Illumina; San Diego, CA, USA; RRID:SCR_016379]. The genomic reference sequence used was genome GRch37/hg19. The following software tools were utilized in the experimental setup and data analysis: Illumina Experiment Manager 1.6.0 (Illumina; San Diego, CA, USA), MiSeq Control Software 2.4 (Illumina; San Diego, CA, USA), Real Time Analysis 1.18.54 (Illumina; San Diego, CA, USA), Sequence Analysis Viewer 1.8.37 (Illumina; San Diego, CA, USA), MiSeq Reporter 2.5.1 (Illumina; San Diego, CA, USA), and SureCall 3.0.1.4 (Agilent Technologies; Santa Clara, CA, USA). A minimum of 80% reads at quality scores of AQ30 or higher were required to pass quality control. The lower limit of detection of this assay (analytical sensitivity) for single nucleotide variations was determined to be 5% (one mutant allele in the background of nineteen wild type alleles) to 10% (one mutant allele in the background of nine wild type alleles). Testing of patients with active hematologic malignancies was limited to somatic mutations only.

**Analysis of epigenetic state in AML cells *in vitro***. We determined the H3K27Ac status in untreated and Menin inhibitor-treated MOLM13 cells by ChIPmentation following a previously described protocol (2), ChIP-Seq libraries were generated with a Nextera DNA Library Preparation Kit containing the mutant Tn5 transposase (Illumina, San Diego, CA; Catalog number: FC-121-1030). The DNA fragments were indexed utilizing a Nextera Index Kit (Illumina, San Diego, CA; Catalog number: FC-121-1011) and amplified by PCR utilizing NEBNext® High-Fidelity 2X PCR Master Mix according to the manufacturer’s protocol (New England Biolabs, Ipswich, MA). Library fragments were amplified for 12-15 cycles utilizing the denaturation, annealing, and extension times as previously described (2). The amplified library fragments were PCR-purified with a Qiagen MinElute column (Qiagen, Germantown, MD) then size selected with a 0.65X bead volume to remove large fragments (remaining on the beads), then the supernatant was combined with a 1.0X SPRI bead volume to remove fragments shorter than 200 bp. Library fragments were incubated with AMPure XP SPRI beads (Beckman Coulter, Indianapolis, IN) for 10 minutes at room temperature in 1.5 mL microcentrifuge tubes. The mixture was placed on a magnetic stand for 10 minutes. The supernatant was removed and the SPRI beads were washed twice with fresh 80% ethanol (30 seconds each wash) and air-dried for 2-3 minutes. Library DNA was eluted from the SPRI beads with a 20 µL volume of 10 mM Tris-HCl (pH 8.5). Beads were incubated at room temperature for 10 minutes, then the tubes were transferred to a magnetic stand for 10 minutes. The supernatant containing the DNA libraries was carefully removed by pipetting and transferred into a clean microcentrifuge tube. The individual libraries were quantified by Thermo Fisher Qubit [Thermo Fisher Qubit fluorimeter, RRID:SCR_018095] fluorometric quantification and quality checked by Agilent Bioanalyzer 2100 [Agilent 2100 Bioanalyzer Instrument, RRID:SCR_019389] analysis, respectively. Individual libraries were pooled into one tube, purified over a Qiagen MinElute column [QIAGEN, RRID:SCR_008539], eluted in 20 µL of 10 mM Tris, (pH 8.5) and sequenced on a NextSeq 500 next generation sequencer (Illumina NextSeq 500, RRID:SCR_014983) utilizing a 150 cycle high-output kit (Illumina, San Diego, CA). Raw sequencing data was mapped using TopHat2 (3) [TopHat, RRID:SCR_013035] onto the human genome build UCSC hg38 (NCBI 51) for human data and log2-fold changes were calculated with diffReps (4) [diffReps, RRID:SCR_010873]. Sequence tracks were visualized with IGV software [Integrative Genomics Viewer, RRID:SCR_011793] (5, 6).

**Plasmid Generation, Viral Packaging, and Creation of Cell Lines.** Plasmid constructs for the production of lentivirus were transfected with packaging plasmids psPAX2 and pMD2.G into HEK-293T cells utilizing jetPRIME reagent (PolyPlus Transfection, New York, NY). The psPAX2 and pMD2.G packaging plasmids were a gift from Didier Trono (Addgene plasmid #12260 and #12259 [RRID: Addgene_12260; RRID: Addgene_12259]). Media was changed the following day. Viral supernatant was collected 72 hours post transfection and filtered through a 0.45 µm PES membrane. AML cells were seeded at 5 x 10^5^ cells/mL in a 50:50 mix of media and lentiviral supernatant with 8 µg/mL polybrene (Sigma-Aldrich). The following day, the viral supernatant was removed by centrifugation and cells were transduced with fresh viral supernatant for an additional 24 hours. To generate luciferase-expressing AML cells, pHIV-Luc-ZsGreen (a gift from Bryan Welm [Addgene plasmid #39196;http://n2t.net/addgene:39196; RRID:Addgene_39196]) was packaged as above and transduced into MOLM13 or patient-derived AML PDX cells. ZsGreen-positive cells were sorted by flow cytometry (FACSAria, FL-1 channel, top 10% brightest GFP-expressing cells), and expanded in culture or grown in NSG mice prior to their utilization in therapeutic in vivo mouse studies. To generate AML cells (MOLM13) with stable expression of SP-Cas9, lentiCas9-Blast (a kind gift from Feng Zhang; Addgene #52962; RRID:Addgene_52962) was utilized. Transduced cells were selected with 8-10 µg/mL of blasticidin for 10 days. Expression of Cas9 in AML cells was confirmed by immunoblot analyses utilizing anti-FLAG M2 (Sigma-Aldrich) and anti-Cas9 antibodies.

**CRISPR/Cas9-mediated gene editing in cultured AML cells.** To study the effects of knockout of Menin in AML cells, the CHOP-CHOP prediction algorithm (7) was utilized to develop guide RNAs. Guide RNAs were developed against exon 2 and exon 6 sequence of Menin. High scoring sgRNAs were synthesized by Synthego, Inc. For MOLM13 cells without stable Cas9 expression, to obtain Cas9-sgRNA RNPs (ribonucleoprotein complexes), 1 μg of synthetic sgRNA was incubated with 1.5 μg recombinant Cas9 protein (Synthego, Inc.) for 15 min at room temperature. MOLM13 cells were transfected by electroporation utilizing the Amaxa 4D-Nucleofector device with a Cell Line-Specific Nucleofector Kit (Amaxa GmbH, Cologne, Germany) as per the manufacturer's instructions. Immediately post-transfection, cells were plated in complete media containing no antibiotics and 2 µM of ROCK inhibitor (Y-27632, Selleck Chemicals; Houston, TX) and allowed to recover for 24 hours. Knockout of Menin was confirmed by mRNA expression (qPCR) and Western blot analysis 5-6 days post transfection. Gene-edited AML cells were treated with venetoclax for 48 hours or abemaciclib for 96 hours and the % of Annexin V-positive, apoptotic cells or To-Pro-3 iodide positive, non-viable cells were determined by flow cytometry.

**Generation of a dTAG13-responsive Menin-FKBP12^F36V^-HA expressing AML cell line.** To generate an in-frame fusion between the Menin cDNA and the F36V mutant FKBP12 cDNA, a Menin cDNA clone (Clone ID:100003387; Catalog ID: OHS5893-202491930) was obtained from Dharmacon/Horizon Discovery (Boulder, CO). The mutant FKBP12 cDNA was amplified from pET15b His6-FKBPF36V which was a gift from Thomas Wandless (Addgene plasmid #73180) [RRID:Addgene_73180]. An attB1 site was incorporated into the N-terminus of the primer for Menin (attB1 Menin.for 5’- GCTT **ACA AGT TTG TAC AAA AAA GCA GGC TTC** ACC ATG GGG GCT GAA GGC CGC-3’) and a *Not1* restriction site into the reverse primer (NotI Menin.rev 5’- GTC A **GCG GCC GC** GAG GCC TTT GCG CTG CC-3’), thus removing the native stop codon. A *Not1* restriction site was designed into the forward primer of the FKBP12 cDNA (NotI FKBP12.for 5’-GTC A **GCG GCC GC**T GGA GTG CAG GTG GAA ACC ATC TC-3’) and a C-terminal HA-tag and attB2 site were designed into the reverse primer (attB2 HA FKBP12.rev 5’-GGGG **AC CAC TTT GTA CAA GAA AGC TGG GTA** TCA AGC GTA ATC TGG AAC ATC GTA TGG GTA AGC GTA ATC TGG AAC ATC GTA TGG GTA TTC CAG TTT TAG AAG CTC CAC ATC GAA GAC-3’). The cDNAs were amplified by PCR using Pfusion polymerase according to the manufacturer’s recommendations for temperature and extension times. PCR products were column-purified and digested with *Not1* overnight at 37°C. The resulting fragments were gel purified and ligated with T4 DNA ligase overnight at 16°C. The pDONR^TM^221 vector (Invitrogen, Carlsbad, CA) was utilized for the BP clonase reaction. The Menin-FKBP12 fusion DNA was introduced into the Gateway cloning vector pDONR^TM^221 following the manufacturer’s BP clonase protocol and incubating the reaction at room temperature for 2 hours. The recombined DNA was transformed into *E. coli* TOP10 cells and selected with 100 µg/mL of ampicillin. Transformants were checked by DNA sequencing. The fusion cDNA was transferred by Gateway cloning into pLEX_305, a kind gift from David Root (Addgene # 41390) [RRID:Addgene_41390] utilizing an LR clonase reaction. The resulting plasmid was transformed into *E. coli* DH5 alpha cells and selected with 100 µg/mL of ampicillin. Positive clones were confirmed by DNA sequencing and then pLEX_305/Menin-FKBP12^(F36V)^-HA vector was combined with packaging vectors (as above) to generate lentiviral particles in HEK293T cells. MOLM13 cells that had been previously engineered to stably express SP-Cas9 (as above) were transduced with lentivirus supernatants and selected with 0.5 µg/mL of puromycin for 96 hours. Next, two splice-blocking sgRNAs were used to knock out the endogenous Menin: one in the intron between exon 3 and exon 4, and one in the intron between exon 5 and exon 6. DNA oligos were synthesized, annealed to each other in annealing buffer, then ligated into digested LRG [Lenti_sgRNA_EFS_GFP] vector, a kind gift from Christopher Vakoc (Addgene #65656) [RRID: Addgene_65656] and transformed into *E. coli* Sure2 cells (Part Number: 200152; Agilent Technologies, Santa Clara, CA). Positive transformants were confirmed by Sanger sequencing, then combined with packaging vectors to generate lentiviral particles. Lentiviral supernatant was combined with MOLM13-Menin-FKBP12^(F36V)^-HA-expressing cells and incubated for 48 hours. GFP-positive cells were selected by FACS sorting and expanded for experimentation. dTAG-13 was synthesized as previously described (8). Cells were treated with dTAG-13 as indicated.

**Assessment of percentage non-viable cells.** Following designated treatments (72-96 hours), cultured cell lines or PD- AML cells, were washed with 1X PBS, stained with TO-PRO-3 iodide (Life Technologies, Carlsbad, CA) and analyzed by flow cytometry on a BD Accuri CFlow-6 flow cytometer (BD Biosciences, San Jose, CA). We used matrix dosing of agents in combinations to allow synergy assessment utilizing the SynergyFinder V2 online web application tool (<http://synergyfinder.fimm.fi/>) and Delta synergy scores by ZIP method (9, 10).

**Assessment of leukemia cell differentiation.** Following treatment with Menin inhibitors, cells were harvested and washed with 1X PBS. Cells were re-suspended in 0.5% BSA/PBS and stained with APC-conjugated anti-CD11b antibody [RRID:AB_398456] or APC-conjugated IgG1 isotype control antibody [RRID:AB_398613] in the dark, at 4°C for 15-20 minutes. Cells were washed with 0.5% BSA/PBS by centrifugation at 125 x g for 5 minutes, and then suspended in 0.5% BSA/PBS for analysis by flow cytometry. Cells were assessed in the FL-4 fluorescence channel on a BD Accuri CFLow6 flow cytometer. Differentiation of leukemia cells was also determined by examination of cellular/nuclear morphology. Cells were cytospun onto glass slides at 500 rpm for 5 minutes. The cytospun cells were fixed and stained with a Protocol® HEMA3 stain set (Fisher Scientific, Kalamazoo, MI). Cellular/nuclear morphology was assessed by light microscopy. Two hundred cells were counted in at least 5 different sections of the slide for each condition. The % morphologic differentiation is reported relative to the control cells. Each experiment was performed at least twice.

**RNA isolation and quantitative polymerase chain reaction.** Following the designated treatments, total RNA was isolated from cultured or patient-derived AML cells utilizing a PureLink RNA Mini kit from Ambion, Inc. (Austin, TX) and reverse transcribed with a High Capacity Reverse Transcription kit from Life Technologies (Carlsbad, CA). Quantitative real-time PCR analysis for the expression of target genes was performed on cDNA using TaqMan probes and a TaqMan Universal PCR Mastermix from Applied Biosystems (Foster City, CA). Relative mRNA expression was normalized to the expression of GAPDH and compared to the untreated cells.

**Cell lysis and protein quantitation.** Untreated or drug-treated cells were centrifuged, and the cell pellets were incubated in lysis buffer on ice for 20 minutes (11). After centrifugation, an aliquot of each cell lysate was diluted 1:10 and the protein content was quantitated using a BCA protein quantitation kit (Pierce, Rockford, IL), according to the manufacturer’s protocol. Protein concentrations were determined by comparing the absorbance at 562 nm compared to a known concentration range of bovine serum albumin (BSA) from 0.125 mg/mL to 2 mg/mL.

**SDS-PAGE and immunoblot analyses.** Seventy-five micrograms of total cell lysate were used for SDS-PAGE. Western blot analyses were performed on total cell lysates using specific antisera or monoclonal antibodies. Blots were washed with 1× PBST, then incubated in IRDye 680RD goat anti-mouse (RRID:AB_10956588) or IRDye 800CW goat anti-rabbit (RRID:AB_621843) secondary antibodies (LI-COR, Lincoln, NE) for 1 h, washed three times in 1× Phosphate Buffered Saline with Tween®20 (PBST) and scanned with an Odyssey CLX Infrared Imaging System utilizing Image Studio 5.0 Software (RRID:SCR_015795) (LI-COR, Lincoln, NE). The expression levels of β-Actin or GAPDH in the cell lysates were used as the loading control for the western blots. Immunoblot analyses were performed at least twice. Representative immunoblots were subjected to densitometry analysis. Densitometry analysis was performed using ImageJ software (12).

**Detection of senescence-associated β-galactosidase expression by flow cytometry**. MOLM13 and OCI-AML3 cells were treated with SNDX-50469 and/or abemaciclib for 48 hours. At the end of incubation, cells were harvested by centrifugation at 125 x g for 5 minutes. Cells were washed with 1X PBS and centrifuged at 125 x g for 5 minutes. The 1X PBS was removed and cells were fixed in 100 µL of 4% paraformaldehyde for 10 minutes at RT. Cells were washed with 1% BSA/PBS at 500 x g for 5 minutes and aspirated. Cells were suspended in 100 µL of Working Solution (Cell Event^TM^ Senescence Green Flow Cytometry Assay Kit, Catalog # C10841) and incubated at 37°C (with no CO_2_) for 90 minutes. Cells were washed with 1% BSA/PBS at 500 x g for 5 minutes and aspirated. Cells were suspended in 200 µL of 1% BSA/PBS and analyzed by flow cytometry utilizing the FL-1 fluorescence channel on a BD-Accuri CFlow6 flow cytometer.

**In vivo models of de novo AML:** All in vivo studies were approved by and conducted in accordance with the guidelines of the IACUC at the M.D. Anderson Cancer Center, an AAALAC-accredited facility. Male and female NOD.Cg-Prkdc^scid^ Il2rg^tm1Wjl^/SzJ (NSG) mice (stock number: 005557; 4-6 weeks of age) [Jackson Labs, Bar Harbor, ME; RRID: IMSR_JAX:005557] were exposed to 2.5 Gy of gamma radiation from a Cesium source. The following day, mice (n=10 per cohort) were injected in the lateral tail vein with 0.5 x 10^6^ GFP-luciferase expressing MOLM13 or 3.0 x 10^6^ GFP-luciferase expressing AML PDX (Dana Farber PDX number: DF87153) and monitored for 4-5 days. Mice were imaged utilizing a Xenogen IVIS Lumina in vivo imaging system to document engraftment before treatment was initiated. Mice were randomized into groups based on equivalent mean bioluminescent intensity to control for variation in cell engraftment and variation between different treatment groups. Treatments were initiated on day 5. For the MOLM13 model, mice were treated with SNDX5613 (50 mg/kg, B.I.D., daily x 5 days, by oral gavage) and/or venetoclax (30 mg/kg, daily x 5 days, by oral gavage) for 3 weeks. Mice were imaged weekly by bioluminescent imaging to document treatment efficacy and/or disease progression. Total bioluminescent flux was recorded as photons/second. Mice that became moribund or experienced hind limb paralysis were euthanized according to the approved IACUC protocol. Department of Veterinary Medicine staff members assisting in determining when euthanasia was required were blinded to the experimental conditions of the study. The survival of the mice is represented by a Kaplan-Meier plot. Significance was determined by a Mantel-Cox log rank test. P-values of less than 0.05 were assigned significance. For the AML PDX mouse model, mice were treated with SNDX5613 (75 mg/kg, B.I.D., daily x 5 days, by oral gavage) and/or venetoclax (30 mg/kg, daily x 5 days, by oral gavage) for 4 weeks. All mice in each treatment cohort were imaged utilizing a Xenogen IVIS Lumina in vivo imaging system once per week to monitor disease status and treatment efficacy. Total bioluminescent flux was recorded as photons/second. Mice that became moribund or experienced hind limb paralysis were euthanized according to the approved IACUC protocol. Department of Veterinary Medicine staff members assisting in determining when euthanasia was required were blinded to the experimental conditions of the study. The survival of the mice is represented by a Kaplan-Meier plot. Significance was determined by a Mantel-Cox log rank test. P-values of less than 0.05 were assigned significance. Mice (n=5 per cohort) were also injected by lateral tail vein with 2.0 x 10^6^ MLL-AF9 + FLT3-TKD expressing AML PDX cells (Dana Farber PDX number: DF68555) and monitored for 24 hours prior to starting treatment. For the MLL-AF9 + FLT3-TKD AML PDX mouse model, mice were treated with SNDX5613 (75 mg/kg, B.I.D., daily x 5 days, by oral gavage) for 4 weeks. Mice that became moribund or experienced hind limb paralysis were euthanized according to the approved IACUC protocol. Department of Veterinary Medicine staff members assisting in determining when euthanasia was required were blinded to the experimental conditions of the study. The survival of the mice is represented by a Kaplan-Meier plot. Significance was determined by a Mantel-Cox log rank test. P-values of less than 0.05 were assigned significance.

**Power analysis for in vivo studies**. With a sample size of 10 mice per group, we can achieve 79.5% power to detect a difference of overall survival at a significance level of 0.05 with one-sided log-rank test, assuming 30% of mouse-survival at the end of study in the experimental group.

**Statistical analysis**. Significant differences between values obtained in AML cells treated with different experimental conditions compared to untreated control cells were determined using the Student’s t-test in GraphPad V8. For the *in vivo* mouse models, a two-tailed, unpaired t-test was utilized for comparing total bioluminescent flux. For survival analysis, a Kaplan-Meier plot and a Mantel–Cox log rank test were utilized for comparisons of different cohorts. P-values of < 0.05 were assigned significance.

**Data and Software availability**. ChIP-Seq datasets have been deposited in GEO and assigned accession ID (GSE190719).

**REFERENCES for Supplemental Methods**

1. Khan M, et al. Clinical outcomes and co-occurring mutations in patients with runx1-mutated acute myeloid leukemia. *Int J Mol Sci* **18**, (2017).
2. Schmidl C, Rendeiro AF, Sheffield NC, Bock C. ChIPmentation: fast, robust, low-input ChIP-seq for histones and transcription factors. *Nat Methods* **12**, 963-5, (2015).
3. Kim D, et al. TopHat2: accurate alignment of transcriptomes in the presence of insertions, deletions and gene fusions. *Genome Biol*. **14**, R36, (2013).
4. Shen L, Shao NY, Liu X, Maze I, Feng J, Nestler EJ. diffReps: detecting differential chromatin modification sites from ChIP-seq data with biological replicates. *PLoS One* **8**, e65598 (2013).
5. Robinson JT, et al. Integrative genomics viewer. *Nat Biotechnol* **29**, 24-6, (2011).
6. Thorvaldsdottir H, Robinson JT, Mesirov JP. Integrative Genomics Viewer (IGV): high-performance genomics data visualization and exploration. *Briefings in Bioinformatics* **14**, 178-92, (2013).
7. Labun K, Montague TG, Gagnon JA, Thyme SB, Valen E. CHOPCHOP v2: a web tool for the next generation of CRISPR genome engineering. *Nucleic Acids Res* **44**, W272-6, (2016).
8. Nabet B, et al. The dTAG system for immediate and target-specific protein degradation. *Nat Chem Biol* **14**, 431-441, (2018).
9. Ianevski A, He L, Aittokallio T, Tang J. SynergyFinder: a web application for analyzing drug combination dose-response matrix data. *Bioinformatics* **33**, 2413-2415, (2017).
10. Ianevski, A., Giri, A. K., and Aittokallio, T. SynergyFinder 2.0: visual analytics of multi-drug combination synergies, *Nucleic Acids Res* **48**, W488-W493, (2020).
11. Fiskus W, Verstovsek S, Manshouri T, Rao R, Balusu R, Venkannagari S, et al. Heat shock protein 90 inhibitor is synergistic with JAK2 inhibitor and overcomes resistance to JAK2-TKI in human myeloproliferative neoplasm cells. *Clin Cancer Res* **17**,7347-58, (2011).
12. Schneider CA, Rasband WS, Eliceiri KW. NIH Image to ImageJ: 25 years of image analysis. *Nat Methods* **9**, 671-5, (2012).
